# Trajectories of plant-based dietary patterns and their sex-specific associations with cardiometabolic health among young Australian adults

**DOI:** 10.1186/s12966-025-01765-0

**Published:** 2025-05-27

**Authors:** Laura E. Marchese, Sarah A. McNaughton, Gilly A. Hendrie, Priscila P. Machado, Therese A. O’Sullivan, Lawrence J. Beilin, Trevor A. Mori, Kacie M. Dickinson, Katherine M. Livingstone

**Affiliations:** 1 Institute for Physical Activity and Nutrition (IPAN), School of Exercise and Nutrition Sciences, Deakin University, Geelong, VIC 3220 Australia; 2 Health and Well-Being Centre for Research Innovation, School of Human Movement and Nutrition Sciences, University of Queensland, St Lucia, QLD 4067 Australia; 3 School of Exercise and Nutrition Sciences, Deakin University, Geelong, VIC 3220 Australia; 4 CSIRO Health and Biosecurity, Adelaide, SA 5000 Australia; 5 Nutrition & Health Innovation Research Institute, School of Medical and Health Sciences, Edith Cowan University, Joondalup, WA Australia; 6 Medical School, Royal Perth Hospital Unit, University of Western Australia, Perth, Australia; 7 Caring Futures Institute, College of Nursing and Health Sciences, Flinders University, Adelaide, SA 5001 Australia

**Keywords:** Plant-based diet, Dietary patterns, Cardiometabolic health, Adults, Adolescence, Group-based trajectory modelling

## Abstract

**Background:**

Plant-based diets are associated with favourable cardiovascular health markers. Although increasingly consumed among younger demographics, it is unclear how plant-based diet quality tracks from adolescence to young adulthood, and how this impacts cardiovascular health later in life. Thus, this study aimed to explore trajectories of plant-based dietary patterns from adolescence to young adulthood and investigate associations with cardiometabolic health markers in young Australian adults.

**Methods:**

Longitudinal data from 417 participants from the Raine Study were included. Semi-quantitative food frequency questionnaires conducted at 14, 20, and 27 years of were used to derive three plant-based diet quality index scores: an overall plant-based diet (PDI), a healthy plant-based diet (hPDI), and a less healthy plant-based diet (uPDI). Markers of cardiometabolic health included waist circumference, blood lipids, and blood pressure obtained at 14 and 28 years of age. Group-based trajectory modelling was used to describe plant-based diet quality trajectory groups from adolescence to young adulthood. Multivariate linear regression models were used to investigate associations with cardiovascular health markers.

**Results:**

Plant-based diet quality trajectory groups were different by sex, but remained relatively stable over the life stages, with participants remaining either above or below average diet quality at all time points. Associations with cardiovascular health outcomes differed between the sexes, with the hPDI having the greatest number of associations for females, and the uPDI for males. Being female with a higher hPDI score was associated with lower insulin (β = -1.11 (95% CI -2.12, -0.09)), HOMA-IR (β = -0.25 (95% CI -0.48, -0.01)), systolic blood pressure (β = -2.75 (95% CI -5.31, -0.19)), and hs-CRP (β = -1.53 (95% CI -2.82, -0.23)), and higher HDL-cholesterol (β = 0.13 (95% CI -0.03, 0.23)) compared to females with lower hPDI scores. Being male in the higher scoring uPDI group was associated with higher waist circumference (β = 3.12 (95% CI 0.61, 5.63)), waist-to-height ratio (β = 0.02 (95% CI 0.01, 0.03)), insulin (β = 1.54 (95% CI 0.33, 2.76)), HOMA-IR (β = 0.35 (95% CI 0.07, 0.63)), and hypertension status (β = 6.60 (95% CI 1.04, 42.00)) when compared to the lower scoring uPDI group.

**Conclusions:**

This study provides new insights into how plant-based diets track across adolescence into adulthood, impacting on cardiometabolic risk factors differently for males and females. Findings highlight the importance of early sex-specific interventions in adolescence to reduce future risk of cardiovascular-disease.

**Supplementary Information:**

The online version contains supplementary material available at 10.1186/s12966-025-01765-0.

## Background

Poor cardiometabolic health is typically defined by a cluster of suboptimal modifiable risk factors, including an unhealthy diet, high body weight, smoking, low physical activity, high blood pressure, fasting cholesterol, and fasting blood glucose levels [1]. Identifying cardiometabolic health risk factors in young adulthood helps to prevent progression to cardiovascular disease (CVD) in middle age and older adults. Recent Australian data has shown a rise in the number of adolescents and young adults presenting with multiple risk factors [2, 3]. Young adults are experiencing risk factors for CVD at an early age, which potentially signals a higher burden from CVD in future generations of Australian adults. Moreover, the percentage of 18 to 24-year-olds meeting vegetable intake recommendations has nearly halved—from 5.7% in 2011-12 to 3.0% in 2022 [4, 5]. Dietary patterns low in plant foods, such as fruits, vegetables and legumes, as well as those high in red and processed meat and ultra-processed foods are known to increase risk of poor cardiometabolic health and CVD [6, 7].

Research suggests that people following plant-based diets, characterised by high intake of plant-based foods such as fruits, vegetables, and wholegrains, with little to no animal-sourced foods tend to be younger than omnivores [8–11]. Many adolescents and young adults are adopting plant-based dietary patterns for ethical, environmental and financial reasons [12]. Dietary patterns adopted during adolescence and young adulthood are known to track into later life, impacting on cardiometabolic health and CVD risk [13–15]. However, the many changing circumstances across this life stage, such as moving out of home or starting a family, can impact on the stability of dietary patterns during this time, which may translate to disparities in the development of poor cardiometabolic health in young adulthood. Therefore, there is a need to examine trajectories of plant-based dietary patterns across this formative life stage.

Despite emergence of studies using plant-based diet quality scores, most of these studies are cross-sectional, and longitudinal studies have used cohort data of older populations [16] and not adolescents or younger adults. Additionally, previous research has identified that sex-specific dietary patterns influence cardiovascular disease risk differently in men and women [17]. However, there is a lack of evidence from longitudinal studies in younger age groups stratified by sex, and cross-sectional associations do not reflect the importance of dietary changes over adolescence and into young adulthood. Additionally, few datasets are available which provide longitudinal dietary and health outcome data over this life period [18]. Hence, analysing dietary pattern trajectories in males and females will provide valuable sex-specific insights into how diets affect cardiovascular health over time. Thus, the aim of this study was to explore trajectories of plant-based dietary patterns from adolescence to young adulthood and investigate associations with cardiometabolic health markers in young Australian adults.

## Methods

### Study design and participants

This study was a secondary analysis of existing data from the Raine Study, details of which have been previously reported [19]. Briefly, the Raine Study is a multigenerational cohort study which started with 2900 pregnant people (referred to as Generation 1 (Gen1)) who were recruited in Perth, Western Australia from 1989 to 1991. Gen 1 participants gave birth to 2868 children (Generation 2 (Gen2)), who have been followed at regular intervals since. Written and informed consent was obtained from the Gen1 parent until their child (Gen2) turned 18 years of age, at which point they provided their own consent. The follow-ups included assessment of dietary intake, body composition, cardiovascular and metabolic parameters, musculoskeletal health, and socio-economic factors among other measures [19]. This study was reported according to the Strengthening the Reporting of Observational Studies in Epidemiology—Nutritional Epidemiology (STROBE-nut) reporting guidelines [20] (Supplementary Table 1) and the Guidelines for Reporting on Latent Trajectory Studies (GRoLTS) checklist [21] (Supplementary Table 2). Ethics for the Raine Study was approved by the University of Western Australia (reference 2019/RA/4/20/5722).

### Dietary intake

Dietary data was reported by the participants at the Gen2-14, 17, 20, 22, and 27 year follow-ups. A semi-quantitative food frequency questionnaire (FFQ) developed by the Commonwealth Scientific and Industrial Research Organisation (CSIRO) was used at the Gen2-14 and Gen2-17 year follow-ups [22]. This questionnaire assessed food and nutrient intakes by collecting information about consumption frequency and serving size of 227 foods and beverages. From the Gen2-20 year follow-up onwards, a 74-item semi-quantitative Dietary Questionnaire for Epidemiological Studies (DQESV2) FFQ developed by the Cancer Council of Victoria was completed [23] as the CSIRO questionnaire was no longer available. The DQESV2 FFQ collected data on frequency, consumption and serving sizes of foods and beverages and has been determined to be appropriate for use in young adults [23]. As the DQESV2 FFQ did not cover the same range of beverages as the CSIRO FFQ, a semiquantitative beverage questionnaire was also used at the Gen2-20 and Gen2-22 year follow-ups to assess additional beverages. This questionnaire included water, soft drinks, energy drinks, tea, and coffee, while the DQESV2 FFQ included juice, milk, and alcohol. Estimates of serving sizes and consumption frequency were collected. Previous research has shown significant agreement between the FFQs [24, 25]. For the purposes of this study, the FFQs conducted at the Gen2-14, Gen2-20, and Gen2-27 year follow-ups were included for analysis. This provided a six-to-seven-year gap between assessments, which enabled estimates of change over time, and highlighted differences across two distinct life stages of adolescence and adulthood. Diet misreporting was estimated using the Goldberg method and used as a categorical variable [26, 27].

### Plant-based diet quality indices (exposure)

The three plant-based diet indices developed and validated by Satija et al. [28] were used to capture the overall quality of plant-based dietary patterns. These a priori indices calculated compliance to a plant-based diet index (PDI), a healthy plant-based diet index (hPDI), and a less healthy/unhealthy plant-based diet index (uPDI), with the calculation method outlined in Supplementary Table 3. These indices were selected as they positively score plant foods and negatively score animal foods, and also utilise epidemiological evidence to categorise plant foods and beverages as healthy or less healthy [29]. This provides a comprehensive assessment of the diet and accounts for nuances of a plant-based diet. To calculate these indices, the foods and beverages from the FFQ data were classified into 17 food groups that comprised of three categories: healthy plant-based foods, less healthy plant-based foods and animal foods. Vegetable oils were excluded from the calculation as this was not specifically analysed in the DQESV2 FFQ, and they have been excluded in previous studies using these indices [30–35]. Foods and beverages were categorised to the 17 food groups in alignment with the original publication and accounted for classification uniformity across the three FFQ timepoints. To ensure this, foods and beverage were cross-checked across the timepoints to ensure consistent classification. Additionally, classification rules were followed throughout. For example, to assign the FFQ items to the food groups, all grains were assumed to be refined unless otherwise specified, and all solid fats such as butter and margarine were assumed to be animal based. FFQ items such as water, alcohol, chocolate powders, condiments, and added sugars were not included in the plant-based diet quality score calculations as they were not included in the food groups classified by Satija et al. [28]. The intake of each food group (in serves based on the Australian standard serve size [36]) was ranked into sex-specific population-based quintiles and given positive or reverse scores. The PDI was created by giving positive scores to all plant food groups and reverse scores to animal food groups. For positive scoring, participants who belong to the highest quintile of a food group received 5, through to participants below the lowest quintile for a food group who received a score of 1. For reverse scores, those above the highest quintile received a score of 1 and those below the lowest quintile scored 5. The scores from the 17 foods groups were summed to generate a possible overall total between 17 and 85, where higher score indicated closer alignment to a plant-based diet. Scores were presented as continuous values. To reconcile potential differences in the dietary intake methods, the plant-based diet quality index scores were standardised.

### Cardiovascular health (outcomes)

Markers of cardiometabolic health recorded at the Gen2-14 and Gen2-28 year follow-ups were included in the analysis. The outcome measures of interest were: waist circumference (cm), waist-to-height ratio (cm), total cholesterol (mmol/L), high-density lipoprotein (HDL) cholesterol (mmol/L), triglycerides (mmol/L), non-HDL-cholesterol (mmol/L), total cholesterol to HDL cholesterol (TC:HDL) ratio (mmol/L), glucose (mmol/L), insulin (mU/L), Homeostatic Model Assessment for Insulin Resistance (HOMA-IR), systolic and diastolic blood pressure (mmHg), high-sensitivity C-reactive protein (hs-CRP) (mg/L) (all continuous), and a combined pre- hypertension/hypertension status (categorical). Trained research assistants collected height measurements using a stadiometer, and waist measurements were taken using a measuring tape across the belly button [37–40]. Resting blood pressure was taken in a seated position and measured using an inflatable cuff fitted around the right arm [37–40]. Fasting blood samples were taken from the arm, and analysed for total cholesterol, HDL cholesterol, triglycerides, glucose, insulin, and hs-CRP [37–40]. Insulin resistance was measured using HOMA-IR and calculated using the formula (insulin [mU/L] x glucose [mmol/L] / 22.5) [37–40] and established cut-offs used for pre-hypertension/hypertension status (normal range systolic (mmHg) < 140 and/or diastolic (mmHg) < 90, Grade 1 range systolic (mmHg) 140–159 and/or diastolic (mmHg) 90–99) [41].

### Socio-demographic characteristics (covariates)

Directed acyclic graphs were constructed, and existing literature was considered to identify which covariates were appropriate for inclusion in the model (Supplementary Fig. 1). Sociodemographic confounders included parent ethnicity (both parents Caucasian, or other) and maternal education level (tertiary education or not) reported by Gen1 at Gen2-8 year follow-up [42]. Behavioural confounders were collected via a questionnaire at the Gen2-14 year follow-up and comprised of smoking status (smoker or non-smoker), alcohol intake over the past 12 months (yes or no), hormonal contraceptive use (yes or no), and physical activity (low active or active) [43]. Physical activity was categorized using a previously published method which uses information relating to the physical activity completed inside and outside of school hours, with those in the lowest tertile scoring as “low active”, and the remaining tertiles assigned as “active” [26]. Covariates were tested for multicollinearity, and no potential evidence was found.

### Statistical analysis

All Gen2 participants were eligible for inclusion in the analysis. Participants were excluded from the study if they (i) were pregnant at the Gen2-20, or Gen2-27 year follow-ups, or, (ii) did not complete at least two FFQs at the Gen2-14, Gen2-20, or Gen2-27 year follow-up, or, (iii) had hs-CRP values > 10 mg/L and a body mass index < 30 kg/m^2^ at the Gen2-28 year follow-up, or, iv) had missing data for outcomes, exposures, or covariates. The third exclusion criterion has been previously applied as an elevated hs-CRP in combination with a lower body mass index is indicative of acute inflammation [39, 44]. To test for differences between included and excluded participants, t-tests were used for continuous variables and Chi-Square tests used for categorical variables. To address variations in biology, risk factors, and existing evidence, the analysis was disaggregated by sex [45, 46]. Characteristics of the participants included for analysis were reported for all continuous and categorical variables at the Gen2-14 year follow-up, apart from maternal education and ethnicity which were collected at the Gen2-8 year follow-up. Mean and standard error were used to report normally distributed data, and median and interquartile range (IQR) for skewed data. All plant-based diet quality index scores were calculated separately for each sex [47], and were normally distributed. Two samples t-tests were used to determine if there was a difference from the plant-based diet quality scores at the Gen2-14 year follow-up, to the Gen2-27 year follow-up [48].

Group-based trajectory modelling allows these longitudinal dietary patterns to be characterized and classifies individuals into distinct groups based on their dietary changes across a specific timeframe [14]. Group-based trajectory modelling of the plant-based diet index scores (PDI, hPDI, uPDI) were used to classify variation in consumption of the plant-based dietary pattern from the Gen2-14, Gen2-20, and Gen2-27 year follow-ups. To allow for comparisons across the indices, standardisation (z-scores) of total plant-based diet quality index scores was applied before identifying the plant-based diet quality trajectory groups. Censored normal models were used, with a quadratic function of time was used due to the three time points available (age in years) as the independent variable and repeated measurements of plant-based diet quality index scores as the outcome variable. To determine the optimal number of groups for the analysis, models with 2 to 5 groups were tested and compared using: Bayesian information criterion (BIC) and the log Bayes Index, a minimum group membership of 5% for each trajectory group [49, 50]. The final models were chosen based on those with a higher entropy, and least negative BIC, indicating a greater fitting model [51] (Supplementary Table 4).

Crude (unadjusted) and multivariate (adjusted) linear regression models were used to evaluate the associations between trajectories of plant-based diet quality trajectory groups and markers of cardiometabolic health at the Gen2-28 year follow-up, adjusted for age, energy intake, diet misreporting status, socio-demographic characteristics (parent ethnicity, maternal education, smoking status, alcohol intake, hormonal contraceptive use (females only), and physical activity), and cardiometabolic health markers all from the Gen2-14 year follow-up. *P*-values were used to gauge strength of the evidence, with *p* < 0.001 indicating very strong evidence, *p* < 0.01 strong evidence, *p* < 0.05 moderate evidence, and *p* ≥ 0.1 indicating insufficient evidence [52]. Data analysis was performed using STATA survey module (v18, STATA Corp., College Station, TX, USA) [49]. No adjustment was made for multiplicity as this was an exploratory analysis rather than hypothesis driven [53].

### Sensitivity analysis

A sensitivity analysis excluding those who misreported energy across any of the FFQs was conducted to further explore the effect of dietary misreporting on the association [26]. To examine the sensitivity of results to the approach used to handle missing data, a second sensitivity analysis was also explored with multiple imputation using chained equations to impute the missing covariate data (number of imputations = 20).

## Results

Of the 2,868 Gen 2 participants, 2,451 were excluded due to missing data on dietary intake or did not meet the inclusion criteria, resulting in 417 participants (*n* = 201 females, *n* = 216 males) included in the present analysis (Supplementary Fig. 2).

### Participant characteristics at baseline

At the Gen2-14 year follow-up, participants had a mean age of 14 years (SD ± 0.19 females, SD ± 0.18 males), most were non-smokers (80% females, 85% males), with over half having consumed alcohol over the past 12 months (63% females, 56% males) (Tables 1 and 2). Most participants had both Caucasian parents (88% females, 86% males), and mothers without tertiary education (60% females, 62% males). Over half of participants had an active physical activity level (65% females, 54% males), and only 3% of females reported hormonal contraceptive use. Participant characteristics included in the analysis were comparable to those excluded (Supplementary Table 5).

**Table 1:** Characteristics of females by plant-based diet quality trajectory groups at baseline (*n* = 201)

	Overall	PDI		hPDI		uPDI	
		Group 1 Low	Group 2 High	Group 1 Low	Group 2 High	Group 1 Low	Group 2 High
N (%)	201 (100.00)	167 (83.08)	34 (16.90)	109 (54.23)	92 (45.77)	95 (47.26)	106 (52.74)
Age (years), mean (± SD)	14.10 (± 0.19)	14.01 (± 0.18)	14.13 (± 0.22)	14.08 (± 0.17)	14.11 (± 0.20)	14.11 (± 0.20)	14.10 (± 0.17)
Maternal education ^a^							
Tertiary education	80 (39.80)	66 (39.52)	14 (41.18)	41 (37.61)	39 (42.39)	48 (50.53)	32 (30.19)
No tertiary education	121 (60.20)	101 (60.48)	20 (58.82)	68 (62.39)	53 (57.61)	47 (49.47)	74 (69.81)
Ethnicity ^a^							
Both parents Caucasian	176 (87.56)	147 (88.02)	29 (85.29)	94 (86.24)	82 (89.13)	83 (87.37)	93 (87.74)
Other	25 (12.44)	20 (11.98)	5 (14.71)	15 (13.76)	10 (10.87)	12 (12.63)	13 (12.26)
Smoking status							
No	161 (80.10)	136 (81.44)	25 (73.53)	83 (76.15)	78 (84.78)	77 (81.05)	84 (79.25)
Yes	40 (19.90)	31 (18.56)	9 (26.47)	26 (23.85)	14 (15.22)	18 (18.95)	22 (20.75)
Alcohol ^b^							
No	74 (36.82)	61 (36.53)	13 (38.24)	45 (41.28)	29 (31.52)	36 (37.89)	38 (35.85)
Yes	127 (63.18)	106 (63.47)	21 (61.76)	64 (58.72)	63 (68.48)	59 (62.11)	68 (64.15)
Contraceptive use							
No	195 (97.01)	163 (97.60)	32 (94.12)	106 (97.25)	89 (96.74)	92 (96.84)	103 (97.17)
Yes	6 (2.99)	4 (2.40)	2 (5.88)	3 (2.75)	3 (3.26)	3 (3.16)	3 (2.83)
Physical activity ^c^							
Low active	71 (35.32)	60 (35.93)	11 (32.35)	39 (35.78)	32 (34.78)	29 (30.53)	42 (39.62)
Active	130 (64.68)	107 (64.07)	23 (67.65)	70 (64.22)	60 (65.22)	66 (69.47)	64 (60.38)
Energy intake (kJ), median (IQR)	8245 (6839–10156)	8046 (6558–10156)	8682 (7383–10218)	8706 (7146–10700)	7896 (6255–9409)	8646 (7070–10218)	7891 (6335–10106)
Plant-based diet quality index scores, mean (± SD)							
Gen2-14 year follow-up ^d^		48.19 (± 4.90)	54.35 (± 3.99)	47.03 (± 7.78)	52.44 (± 6.45)	48.41 (± 6.47)	56.67 (± 5.97)
Gen2-20 year follow-up (*n* = 180) ^e^		49.73 (± 4.85)	58.61 (± 4.69)	46.48 (± 5.49)	58.82 (± 6.02)	46.93 (± 5.00)	56.11 (± 5.89)
Gen2-27 year follow-up (*n* = 176) ^f^		49.14 (± 5.98)*	57.56 (± 5.16)	47.10 (± 6.24)*	58.08 (± 6.14)	45.48 (± 5.55)*	55.96 (± 5.94)*
Diet misreporting ^g^	24 (11.94)	19 (11.38)	5 (14.71)	10 (9.17)	14 (15.22)	12 (12.63)	12 (11.32)
Waist circumference (cm), median (IQR)	71.55 (67.15–78.10)	71.55 (67.45–77.75)	71.40 (65.80–82.00)	72.00 (68.00-80.75)	70.50 (66.38–76.35)	69.65 (66.35–76.25)	72.00 (68.25–80.50)
Waist-to-height ratio (cm), median (IQR)	0.44 (0.41–0.48)	0.44 (0.41–0.48)	0.44 (0.40–0.50)	0.44 (0.41–0.49)	0.44 (0.41–0.47)	0.43 (0.40–0.48)	0.44 (0.42–0.49)
Total cholesterol (mmol/L), mean (± SD)	4.28 (± 0.69)	4.28 (± 0.71)	4.28 (± 0.61)	4.18 (± 0.73)	4.40 (± 0.63)	4.31 (± 0.62)	4.26 (± 0.75)
HDL-cholesterol (mmol/L), mean (± SD)	1.45 (± 0.35)	1.45 (± 0.35)	1.47 (± 0.35)	1.40 (± 0.31)	1.51 (± 0.38)	1.46 (± 0.34)	1.44 (± 0.36)
Triglycerides (mmol/L), median (IQR)	0.95 (0.74–1.16)	0.95 (0.73–1.16)	0.94 (0.80–1.25)	0.91 (0.72–1.14)	0.95 (0.77–1.25)	0.92 (0.75–1.15)	0.96 (0.73–1.21)
Non-HDL-C (mmol/L), mean (± SD)	2.83 (± 0.70)	2.83 (± 0.72)	2.80 (± 0.60)	2.78 (± 0.73)	2.89 (± 0.65)	2.84 (± 0.65)	2.82 (± 0.74)
TC:HDL ratio (mmol/L), median (IQR)	3.01 (2.46–3.53)	3.00 (2.46–3.56)	3.05 (2.44–3.23)	3.02 (2.54–3.53)	2.46 (2.46–3.48)	3.02 (2.44–3.57)	2.99 (2.54–3.47)
Glucose (mmol/L), mean (± SD)	4.72 (± 0.39)	4.71 (± 0.40)	4.78 (± 0.33)	4.75 (± 0.41)	4.69 (± 0.37)	4.76 (± 0.42)	4.68 (± 0.36)
Insulin (mU/L), median (IQR)	10.40 (7.90–13.60)	10.50 (7.80–13.60)	10.30 (7.97–13.90)	10.70 (8.40–14.90)	10.35 (7.60-13.05)	10.50 (7.70–13.40)	10.40 (8.02–13.90)
HOMA-IR, median (IQR)	2.15 (1.64–2.90)	2.17 (1.63–2.90)	2.07 (1.67–3.29)	2.21 (1.70–3.16)	2.13 (1.54–2.73)	2.22 (1.60–2.90)	2.14 (1.66–2.92)
Systolic blood pressure (mmHg), mean (± SD)	107.63 (± 10.04)	107.63 (± 10.02)	107.68 (± 10.25)	107.95 (± 10.34)	107.26 (± 9.71)	107.21 (± 10.37)	108.02 (± 9.76)
Diastolic blood pressure (mmHg), mean (± SD)	58.59 (± 7.13)	58.73 (± 7.04)	57.90 (± 7.60)	58.88 (± 6.98)	58.25 (± 7.33)	58.14 (± 7.53)	59.00 (± 6.76)
hs-CRP (mg/L), median (IQR)	0.22 (0.17–0.68)	0.22 (0.17–0.72)	0.22 (0.17–0.43)	0.29 (0.17–0.86)	0.18 (0.17–0.50)	0.19 (0.17–0.62)	0.25 (0.17–0.72)
Combined pre- hypertension/hypertension status ^1, h^							
Normal	201 (100.00)	167 (100.00)	34 (100.00)	109 (100.00)	92 (100.00)	95 (100.00)	106 (100.00)

All values are n (%) unless otherwise specified. PDI: plant-based diet index, hPDI: healthy plant-based diet index, uPDI: less healthy plant-based diet index, SD: standard deviation, IQR: interquartile range, ^a^ Collected at Gen1-8 year follow-up, ^b^ alcohol in past 12 months, ^c^ physical activity completed inside and outside of school hours, ^d^ Overall: PDI: 49.23 (± 5.28), hPDI: 49.51 (± 7.68), uPDI: 52.77 (± 7.45), ^e^ Overall: PDI: 51.26 (± 5.87), hPDI: 52.24 (± 8.42), uPDI: 51.83 (± 7.15), ^f^ Overall: PDI: 50.67 (± 6.67)*, hPDI: 52.47 (± 8.27)***, uPDI: 50.90 (± 7.78)*, ^g^ Goldberg cutoffs, ^h^ Normal range systolic (mmHg) < 140 and/or diastolic (mmHg) < 90 *indicates *p* < 0.001 difference from Gen2-14 year follow-up plant-based diet quality index score, ** indicates *p* < 0.01 difference from Gen2-14 year follow-up plant-based diet quality index score, *** indicates *p* < 0.05 difference from Gen2-14 year follow-up plant-based diet quality index score

**Table 2:** Characteristics of males by plant-based diet quality trajectory groups at baseline (*n* = 216)

	Overall	PDI		hPDI		uPDI	
		Group 1 Low	Group 2 High	Group 1 Low	Group 2 High	Group 1 Low	Group 2 High
N (%)	216 (100)	76 (35.19)	140 (64.81)	108 (50.00)	108 (50.00)	123 (56.94)	93 (43.06)
Age (years), mean (± SD)	14.10 (± 0.18)	14.10 (± 0.18)	14.10 (± 0.19)	14.07 (± 0.19)	14.10 (± 0.18)	14.06 (± 0.20)	14.10 (± 0.16)
Maternal education ^a^							
Tertiary education	92 (42.59)	27 (35.53)	65 (46.43)	43 (39.81)	49 (45.37)	59 (47.97)	33 (35.48)
No Tertiary education	124 (57.41)	49 (64.47)	75 (53.57)	65 (60.19)	59 (54.63)	64 (52.03)	60 (64.52)
Ethnicity ^a^							
Both parents Caucasian	190 (87.96)	63 (82.89)	127 (90.71)	94 (87.04)	96 (88.89)	106 (86.18)	84 (90.32)
Other	26 (12.04)	13 (17.11)	13 (9.29)	14 (12.96)	12 (11.11)	17 (13.82)	9 (9.68)
Smoking status							
No	183 (84.72)	59 (77.63)	124 (88.57)	90 (83.33)	93 (86.11)	104 (84.55)	79 (84.95)
Yes	33 (15.28)	17 (22.37)	16 (11.43)	18 (16.67)	15 (13.89)	19 (15.45)	14 (15.05)
Alcohol ^b^							
No	96 (44.44)	31 (40.79)	65 (46.43)	44 (40.74)	52 (48.15)	50 (40.65)	46 (49.46)
Yes	120 (55.56)	45 (59.21)	75 (53.57)	64 (59.26)	56 (51.85)	73 (59.35)	47 (50.54)
Physical activity ^c^							
Low active	100 (46.30)	31 (40.79)	69 (49.29)	42 (38.89	58 (53.70)	54 (43.90)	46 (49.46)
Active	116 (53.70)	45 (59.21)	71 (50.71)	66 (66.11)	50 (46.30)	69 (56.10)	47 (50.54)
Energy intake (kj), median (IQR)	9942 (8364–12025)	9550 (7597–11219)	10,178 (8700–12284)	11,274 (9380–13092)	9320 (7568–10448)	10,084 (8532–12284)	9932 (8210–11856)
Plant-based diet quality index scores, mean (± SD)							
Gen-2 14 year follow-up ^d^		45.17 (± 5.00)	51.31 (± 5.61)	44.74 (± 6.42)	54.14 (± 6.52)	49.55 (± 5.93)	57.27 (± 6.30)
Gen-2 20 year follow-up (*n* = 192) ^e^		44.14 (± 4.51)	54.42 (± 4.80)	46.77 (± 6.14)	56.50 (± 6.33)	47.84 (± 5.54)	56.89 (± 4.89)
Gen-2 27 year follow-up (*n* = 201) ^f^		47.66 (± 5.35)**	53.28 (± 5.63)**	47.65 (± 5.65)**	57.11 (± 6.05)*	46.90 (± 6.17)**	57.19 (± 5.86)
Diet misreporting ^g^	16 (7.41)	9 (11.84)	7 (5.00)	2 (1.85)	14 (12.96)	11 (8.94)	5 (5.38)
Waist circumference (cm), median (IQR)	72.50 (68.00-79.20)	71.75 (68.00-78.35)	72.88 (68.18–79.53)	71.75 (67.65–78.58)	73.40 (68.60–79.60)	73.00 (68.25–79.25)	72.00 (67.50–79.00)
Waist-to-height ratio (cm), median (IQR)	0.44 (0.41–0.48)	0.44 (0.42–0.48)	0.43 (0.41–0.48)	0.43 (0.41–0.47)	0.44 (0.42–0.49)	0.44 (0.41–0.49)	0.44 (0.41–0.48)
Total cholesterol (mmol/L), mean (± SD)	4.09 (± 0.66)	4.14 (± 0.65)	4.06 (± 0.67)	4.05 (± 0.70)	4.14 (± 0.62)	4.16 (± 0.69)	4.01 (± 0.61)
HDL-cholesterol (mmol/L), mean (± SD)	1.39 (± 0.30)	1.38 (± 0.36)	1.40 (± 0.27)	1.40 (± 0.36)	1.39 (± 0.27)	1.41 (± 0.32)	1.37 (± 0.29)
Triglycerides (mmol/L), median (IQR)	0.78 (0.61–1.03)	0.78 (0.63–1.06)	0.78 (0.61–1.01)	0.76 (0.59–0.97)	0.81 (0.65–1.12)	0.79 (0.65–1.03)	0.76 (0.60–1.01)
Non-HDL-C (mmol/L), mean (± SD)	2.70 (± 0.66)	2.77 (± 0.68)	2.66 (± 0.64)	2.65 (± 0.71)	2.75 (± 0.60)	2.75 (± 0.70)	2.63 (± 0.59)
TC:HDL ratio (mmol/L), median (IQR)	2.97 (2.53–3.41)	3.04 (2.51–3.79)	2.93 (2.53–3.34)	2.93 (2.40–3.53)	3.01 (2.64–3.38)	2.97 (2.56–3.42)	2.94 (2.46–3.41)
Glucose (mmol/L), mean (± SD)	4.84 (± 0.36)	4.87 (± 0.30)	4.82 (± 0.39)	4.80 (± 0.36)	4.87 (± 0.36)	4.82 (± 0.37)	4.85 (± 0.36)
Insulin (mU/L), median (IQR)	9.03 (6.30-12.45)	9.55 (6.83–12.85)	8.51 (6.19–12.10)	8.50 (6.19–11.95)	9.20 (6.70-12.65)	9.05 (6.30–12.30)	9.00 (6.30–12.60)
HOMA-IR, median (IQR)	1.97 (1.33–2.82)	2.01 (1.42–2.91)	1.89 (1.28–2.69)	1.77 (1.31–2.76)	2.06 (1.45–2.82)	1.98 (1.32–2.72)	1.86 (1.34–2.90)
Systolic blood pressure (mmHg), median (IQR)	113.50 (106.75–121.00)	114.50 (106.75-121.75)	113.50 (106.75-120.75)	113.00 (106.25-121.25)	114.00 (107.00-121.00)	114.00 (108.00-121.00)	112.50 (106.00-121.00)
Diastolic blood pressure (mmHg), mean (± SD)	58.82 (± 7.38)	57.53 (± 7.25)	59.05 (± 7.42)	58.82 (± 7.06)	58.21 (± 7.70)	58.51 (± 7.50)	58.53 (± 7.25)
hs-CRP (mg/L), median (IQR)	0.23 (0.17–0.66)	0.23 (0.17–0.57)	0.23 (0.17–0.76)	0.23 (0.17–0.59)	0.22 (0.17–0.80)	0.25 (0.17–0.61)	0.20 (0.17–0.70)
Combined pre- hypertension/hypertension status ^1, h^							
Normal	211 (97.69)	74 (97.37)	137 (97.86)	106 (98.15)	105 (97.22)	118 (95.93)	93 (100.00)
Grade 1 (mild) hypertension	5 (2.3)	2 (2.63)	3 (2.14)	2 (1.85)	3 (2.78)	5 (4.07)	0 (0.00)

All values are n (%) unless otherwise specified. PDI: plant-based diet index, hPDI: healthy plant-based diet index, uPDI: less healthy plant-based diet index, SD: standard deviation, IQR: interquartile range, ^a^ Collected at Gen1-8 year follow-up, ^b^ alcohol in past 12 months, ^c^ physical activity completed inside and outside of school hours, ^d^ Overall: PDI: 49.15 (± 6.14), hPDI: 49.44 (± 7.99), uPDI: 52.88 (± 7.18), ^e^ Overall: PDI: 50.99 (± 6.75), hPDI: 51.84 (± 7.90), uPDI: 51.76 (± 6.91), ^f^ Overall: PDI: 51.24 (± 6.15)*, hPDI: 52.26 (± 7.52)*, uPDI: 51.41 (± 7.91)***, ^g^ Goldberg cutoffs, ^h^ Normal range systolic (mmHg) < 140 and/or diastolic (mmHg) < 90, Grade 1 range systolic (mmHg) 140–159 and/or diastolic (mmHg) 90–99. *indicates *p* < 0.001 difference from Gen2-14 year follow-up plant-based diet quality index score, ** indicates *p* < 0.01 difference from Gen2-14 year follow-up plant-based diet quality index score, *** indicates *p* < 0.05 difference from Gen2-14 year follow-up plant-based diet quality index score

From the Gen2-14 to the Gen2-27 year follow-ups, mean plant-based diet quality scores for females increased, with a PDI of 49.23 (SD ± 5.28) at the Gen2-14 year follow-up increasing to 51.26 (SD ± 5.87) and 50.67 (SD ± 6.67) at the Gen2-20 and Gen2-27 year follow-ups, respectively (possible range: 17 to 85) (Supplementary Table 6). Scores for the hPDI (14y: 49.51 (SD ± 7.68), 20y: 52.24 (SD ± 8.42), 27y: 52.47 (SD ± 8.27)) increased over time, while uPDI scores decreased (14y: 52.77 (SD ± 7.45), 20y: 51.83 (SD ± 7.15), 27y: 50.90 (± 7.78)). For males, mean PDI (14y: 49.15 (SD ± 6.14), 20y: 50.99 (SD ± 6.75), 27y: 51.24 (SD ± 6.15)) and hPDI (14y: 49.44 (SD ± 7.99), 20y: 51.84 (SD ± 7.90), 27y: 52.26 (SD ± 7.52)) scores increased over time, while uPDI scores decreased (14y: 52.88 (SD ± 7.18), 20y: 51.76 (SD ± 6.91), 27y: 51.41 (SD ± 7.91)) (Supplementary Table 6). There was a statistically significant difference between the plant-based diet quality scores from the Gen2-14 to the Gen2-27 year follow-up for both males and females.

### Trajectories of plant-based dietary patterns

**Fig. 1:**
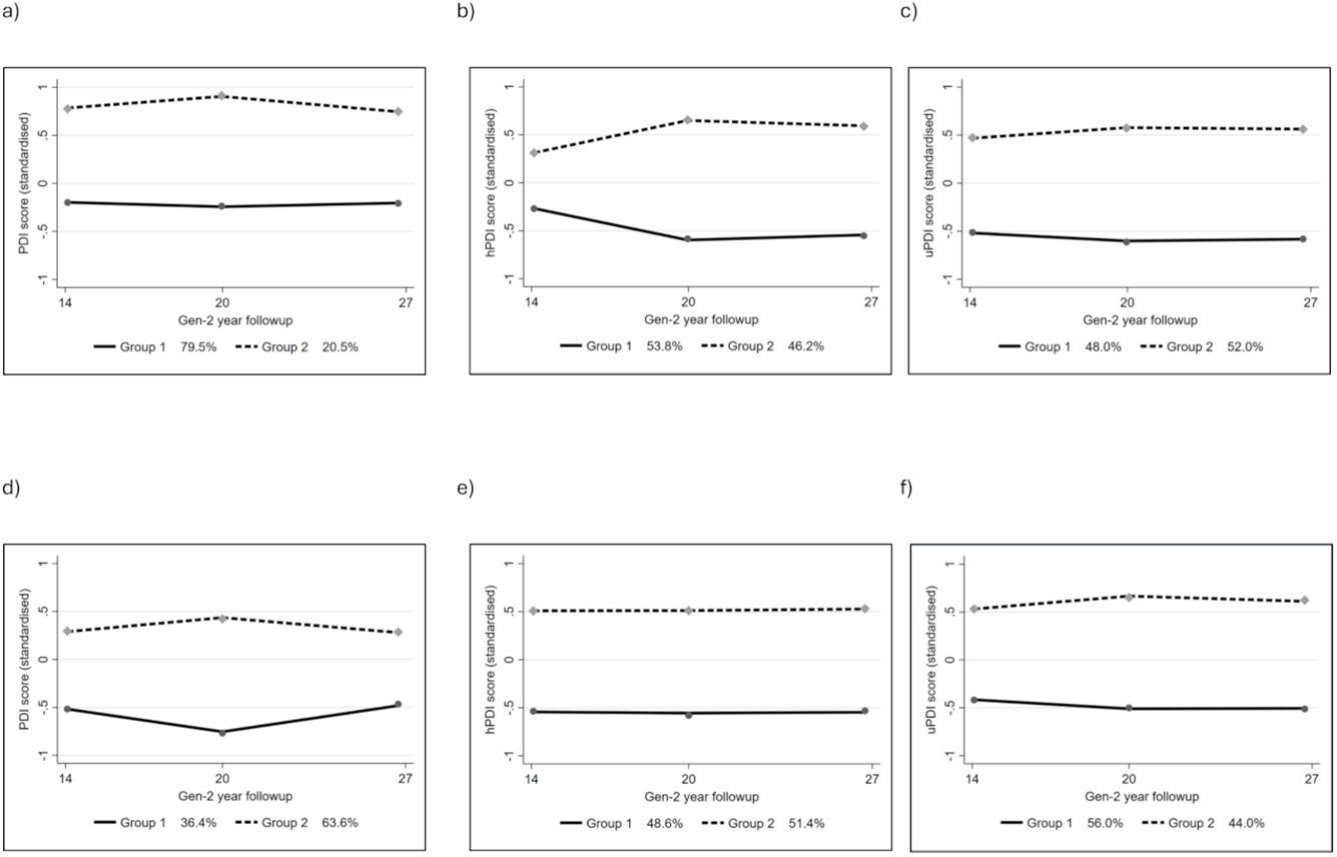
Plant-based diet quality trajectory groups from Gen2-14 to Gen2-27 year follow-ups. (**a**) Females and PDI; (**b**) Females and hPDI; (**c**) Females and uPDI; (**d**) Males and PDI; (**e**) Males and hPDI; (**f**) Males and uPDI

After assigning people to their most probable plant-based diet quality trajectory groups, the percentages of people in each Group (Tables 1 and 2) were similar to the latent probabilities in Fig. 1.

#### Females

Two trajectory groups were identified for each plant-based diet quality index, differentiating low (Group 1) and high (Group 2) diet quality scores. Group 1 of the PDI (83.08%) had a slight but statistically significant increase in diet quality score over time (Fig. 1) (14y: 48.19 (SD ± 4.90), 27y: 49.14 (± 5.98)), but remained below-average throughout. PDI Group 2 participants (16.90%) had an above-average score at the Gen2-14 year follow-up (54.35 (SD ± 3.99)), and a moderate increase over time (27y: 57.56 (SD ± 5.16)). For the hPDI, Group 1 participants (54.23%) had a slight increase in score over time (14y: 47.03 (SD ± 7.78)), 27y: 47.10 (± 6.24)), remaining below average at all time points. Group 2 (45.77%) had an increase in score over time (14y: 52.44 (SD ± 6.45), 27y: 58.08 (± 6.14)) with the highest scores across all groups at the Gen2-20 and Gen2-27 year follow-ups, remaining above average throughout. Contrastingly, both uPDI groups has statistically significant decreases over time. Group 1 (47.26%) scored below average throughout (14y: 48.41 (SD ± 6.47), 27y: 45.48 (SD ± 5.55)). Group 2 (52.74%) had the highest initial score across all trajectory groups (14y: 56.67 (SD ± 5.97) 27y: 55.96 (SD ± 5.94)) and consistently scored above average.

#### Males

For males, two trajectory groups were also identified for each plant-based diet quality index. PDI Group 1 (35.19%) participants had a slight increase in their score over time (14y: 45.17 (SD ± 5.00), 27y: 47.66 (SD ± 5.35)) but remaining below-average throughout. Group 2 of the PDI (64.81%) and had a small score increase over time (14y: 51.31 (SD ± 5.61), 27y: 53.28 (SD ± 5.63)) remaining above average. Both PDI and hPDI groups had statistically significant changes in their score over time. Group 1 participants in the hPDI (50.00%) had below average scores which increased slightly over time (14y: 44.74 (SD ± 6.42), 27y: 47.65 (SD ± 5.65)), while Group 2 (50.00%) had above average scores that increased over time (14y: 54.14 (SD ± 6.52), 27y: 57.11 (SD ± 6.05)). Both uPDI had decreases in their score over time, with Group 1 (56.94%) participants having a statistically significant decrease (14y: 49.55 (SD ± 5.93), 27y: 46.90 (SD ± 6.17)), remaining consistently below average. Group 2 (43.06%) remaining above average throughout (14y: 57.27 (SD ± 6.30), 27y: 57.19 (SD ± 5.86)), and consistently had the highest scores across all the Gen2-14, Gen2-20, and Gen2-27 year follow-ups.

### Trajectories of plant-based dietary patterns and cardiometabolic health

Supplementary tables 7 and 8 present the crude (unadjusted) models, and Tables 3 and 4 present the multivariate (adjusted) linear regression models.

#### Females

In the crude analysis, there was an association between the PDI and hPDI plant-based diet quality trajectory groups with several cardiometabolic health measurements at the Gen2-28 year follow-up (Supplementary Table 7). After adjusting for potential confounders (Table 3), there was an association between the hPDI and uPDI trajectory groups with several cardiometabolic health measurements. The Group 2 hPDI (higher scoring) trajectory group was inversely associated with insulin (β = -1.11 (95% CI -2.12, -0.09)), HOMA-IR (β = -0.25 (95% CI -0.48, -0.01)), systolic blood pressure (β = -2.75 (95% CI -5.31, -0.19)), and hs-CRP (β = -1.53 (95% CI -2.82, -0.23)), and positively associated with HDL-cholesterol (β = 0.13 (95% CI -0.03, 0.23)) when compared to Group 1 (lower scoring). Additionally, the association for waist-to-height ratio approached statistical significance (*p*-value 0.053). The Group 2 uPDI trajectory group was positively associated with triglycerides (β = 0.25 (95% CI 0.10, 0.40)), TC:HDL ratio (β = 0.20 (95% CI 0.00, 0.40), and hs-CRP (β = 1.53 (95% CI 0.24, 2.83)) when compared to Group 1. No strong evidence was found between the PDI trajectory groups and the cardiometabolic health measurements.

**Table 3:** Associations between female plant-based diet quality trajectory groups and markers of cardiometabolic health at the Gen2-28 year follow-up (*n* = 201)

	PDI			hPDI			uPDI		
	Group 1 Low	Group 2 High	*P* value	Group 1 Low	Group 2 High	*P* value	Group 1 Low	Group 2 High	*P* value
Waist circumference	Reference	1.47 (-2.60, 5.34)	0.477	Reference	-2.96 (-6.09, 0.17)	0.063	Reference	2.96 (-0.18, 6.10)	0.064
Waist-to-height ratio	Reference	0.01 (-0.02, 0.04)	0.462	Reference	-0.02 (-0.04, 0.00)	0.053	Reference	0.02 (-0.00, 0.04)	0.065
Total cholesterol	Reference	0.07 (-0.19, 0.33)	0.613	Reference	0.19 (-0.01, 0.40)	0.061	Reference	0.09 (-0.11, 0.29)	0.372
HDL-cholesterol	Reference	0.01 (-0.12, 0.14)	0.922	Reference	0.13 (-0.03, 0.23)	0.010*	Reference	-0.07 (-0.17, 0.03)	0.163
Triglycerides	Reference	0.13 (-0.07, 0.33)	0.195	Reference	0.04 (-0.11, 0.19)	0.612	Reference	0.25 (0.10, 0.40)	0.001*
Non-HDL-C	Reference	0.06 (-0.18, 0.30)	0.614	Reference	0.06 (-0.12, 0.25)	0.518	Reference	0.16 (-0.02, 0.35)	0.085
TC:HDL ratio	Reference	0.03 (-0.23, 0.29)	0.799	Reference	-0.06 (-0.26, 0.14)	0.538	Reference	0.20 (0.00, 0.40)	0.047*
Glucose	Reference	0.16 (-0.50, 0.38)	0.136	Reference	-0.10 (-0.26, 0.07)	0.251	Reference	-0.12 (-0.29, 0.05)	0.156
Insulin	Reference	0.27 (1.07, 1.61)	0.690	Reference	-1.11 (-2.12, -0.09)	0.033*	Reference	0.96 (-0.06, 1.99)	0.066
HOMA-IR	Reference	0.09 (-0.21, 0.40)	0.541	Reference	-0.25 (-0.48, -0.01)	0.038*	Reference	0.19 (-0.05, 0.42)	0.117
Systolic blood pressure	Reference	-0.04 (-3.43, 3.34)	0.979	Reference	-2.75 (-5.31, -0.19)	0.035*	Reference	0.87 (-1.76, 3.49)	0.516
Diastolic blood pressure	Reference	0.58 (-1.94, 3.10)	0.648	Reference	-1.00 (-2.93, 0.92)	0.305	Reference	0.79 (-1.16, 2.74)	0.426
hs-CRP	Reference	-1.06 (-2.76, 0.63)	0.217	Reference	-1.53 (-2.82, -0.23)	0.021*	Reference	1.53 (0.24, 2.83)	0.021*
Combined pre- hypertension/hypertension status^1^ (*n* = 171)	Reference	0.87 (0.08, 9.63)	0.911	Reference	0.72 (0.11, 4.61)	0.730	Reference	0.76 (0.13, 4.50)	0.765

All values are β coefficients and 95% CI unless otherwise specified. ^1^ OR and 95% CI, PDI: plant-based diet index, hPDI: healthy plant-based diet index, uPDI: less healthy plant-based diet index, adjusted for ethnicity, maternal education, smoking status, alcohol intake over the past 12 months, hormonal contraceptive use, and physical activity, energy intake, and diet misreporting status, * indicates results *p* < 0.05

#### Males

From the crude analysis there was an association between the uPDI trajectory groups with several cardiometabolic health measurements at the Gen2-28 year follow-up (supplementary Table 8). After adjusting for all potential confounders (Table 4), there was an association between the hPDI and uPDI trajectory groups with several cardiometabolic health measurements. Participants in Group 2 of the hPDI had an inverse relationship with diastolic blood pressure (β = -2.01 (95% CI -3.98, -0.05)), and a positive association with total cholesterol (β = 0.26 (95% CI -0.02, 0.50)) when compared to Group 1. For Group 2 uPDI participants, there was a positive association with waist circumference (β = 3.12 (95% CI 0.61, 5.63), waist-to-height ratio (β = 0.02 (95% CI 0.01, 0.03), insulin (β = 1.54 (95% CI 0.33, 2.76), HOMA-IR (β = 0.35 (95% CI 0.07, 0.63), and hypertension status (β = 6.60 (95% CI 1.04, 42.00) when compared to Group 1.

**Table 4:** Associations between male plant-based diet quality trajectory groups and markers of cardiometabolic health at the Gen2-28 year follow-up (*n* = 216)

	PDI			hPDI			uPDI		
	Group 1 Low	Group 2 High	*P* value	Group 1 Low	Group 2 High	*P* value	Group 1 Low	Group 2 Medium	*P* value
Waist circumference	Reference	-2.24 (-4.89, 0.39)	0.094	Reference	-1.54 (-4.21, 1.14)	0.259	Reference	3.12 (0.61, 5.63)	0.015*
Waist-to-height ratio	Reference	-0.01 (-0.03, -0.00)	0.116	Reference	-0.01 (-0.02, 0.01)	0.418	Reference	0.02 (0.01, 0.03)	0.006*
Total cholesterol	Reference	-0.01 (-0.24, 0.23)	0.944	Reference	0.26 (-0.02, 0.50)	0.031*	Reference	0.06 (-0.16, 0.29)	0.575
HDL-cholesterol	Reference	0.00 (-0.07, 0.08)	0.924	Reference	0.05 (-0.03, 0.13)	0.191	Reference	-0.04 (-0.11, 0.03)	0.246
Triglycerides	Reference	0.01 (-0.18, 0.19)	0.955	Reference	0.02 (-0.16, 0.21)	0.794	Reference	0.07 (-0.10, 0.25)	0.414
Non-HDL-C	Reference	0.00 (-0.24, 0.24)	0.999	Reference	0.19 (-0.05, 0.43)	0.111	Reference	0.11 (-0.11, 0.34)	0.326
TC:HDL ratio	Reference	-0.01 (-0.33, 0.31)	0.951	Reference	-0.02 (-0.34, 0.31)	0.914	Reference	0.27 (-0.04, 0.58)	0.084
Glucose	Reference	0.18 (-0.15, 0.51)	0.291	Reference	-0.15 (-0.49, 0.18)	0.370	Reference	0.17 (-0.15, 0.48)	0.299
Insulin	Reference	-0.16 (-1.44, 1.13)	0.810	Reference	-0.08 (-1.38, 1.23)	0.905	Reference	1.54 (0.33, 2.76)	0.013*
HOMA-IR	Reference	-0.03 (-0.32, 0.27)	0.853	Reference	-0.01 (-0.31, 0.28)	0.923	Reference	0.35 (0.07, 0.63)	0.013*
Systolic blood pressure	Reference	-1.04 (-3.60, 1.53)	0.426	Reference	-0.45 (-3.05, 2.14)	0.731	Reference	1.42 (-1.04, 3.87)	0.257
Diastolic blood pressure	Reference	-1.45 (-3.41, 0.50)	0.145	Reference	-2.01 (-3.98, -0.05)	0.044*	Reference	1.33 (-0.54, 3.19)	0.162
hs-CRP	Reference	0.18 (-0.56, 0.92)	0.635	Reference	-0.36 (-1.11, 0.39)	0.341	Reference	0.42 (-0.29, 1.12)	0.242
Combined pre- hypertension/hypertension status^1^ (*n* = 190)	Reference	1.37 (0.27, 6.85)	0.705	Reference	0.89 (0.19, 4.20)	0.880	Reference	6.60 (1.04, 42.00)	0.046*

All values are β coefficients and 95% CI unless otherwise specified. ^1^ OR and 95% CI, PDI: plant-based diet index, hPDI: healthy plant-based diet index, uPDI: less healthy plant-based diet index, adjusted for ethnicity, maternal education, smoking status, alcohol intake over the past 12 months, and physical activity, energy intake, and diet misreporting status, * indicates results *p* < 0.05

### Sensitivity analysis

After participants were excluded for misreporting at any time point, 76 females, and 89 males were included for the sensitivity analysis. After adjusting for potential confounders (Supplementary Table 9), there was an association for females between the uPDI trajectory groups with several cardiometabolic health measurements. Group 2 of the uPDI was positively associated with waist circumference (β = 5.07 (95% CI 0.14, 9.99)), waist-to-heigh ratio (β = 0.03 (95% CI 0.00, 0.06)), total cholesterol (β = 0.44 (95% CI 0.11, 0.77)), triglycerides (β = 0.29 (95% CI 0.06, 0.51)), non-hdl-c (β = 0.49 (95% CI 0.20, 0.78)) and TCHDL ratio (β = 0.39 (95% CI 0.08, 0.71)) when compared to Group 1. As shown in Supplementary Table 10, the analysis did not result in any strong associations being identified between the plant-based diet quality trajectory groups and the cardiometabolic health measurements for males

In the second sensitivity analysis, 248 females and 256 males were included after imputing results for missing covariates (diet misreporting status, smoking status, alcohol intake over the past 12 months, maternal education, and energy intake at baseline). Associations were found for females across the hPDI and uPDI, consistent with the complete case analysis (Supplementary Table 11). Females in Group 2 of the hPDI were associated with HDL-cholesterol and hs-CRP when compared to Group 1, as per the complete case analysis. For females in Group 2 of the uPDI the same association was found for triglycerides as per the complete case analysis, however positive associations were also found for waist-to-height ratio, insulin, and HOMA-IR. Contrary to the complete case analysis, associations were found for males across the PDI and hPDI (Supplementary Table 12). Males in Group 2 of the PDI were negatively associated with waist circumference and waist-to-height ratio compared to Group 1. As per the complete case analysis, males in Group 2 of the hPDI were associated with total cholesterol and diastolic blood pressure, however the multiple imputation analysis also found a positive association with triglycerides.

## Discussion

This study explored trajectories of plant-based dietary patterns from adolescence to young adulthood and investigated associations with cardiometabolic health using data from the Raine Study participants aged 14- to 28-years of age. Three plant-based diet quality indices were used and several different sex-specific trajectory groups for dietary intake were identified. Plant-based diet quality trajectory groups were relatively stable over the life stages and groups remained either above or below average diet quality at all time points. Only the healthy and less healthy plant-based diet quality trajectory groups had associations with cardiometabolic health markers for both males and females. For females, the hPDI exhibited the greatest number of associations with CVD outcomes. Results showed those having higher scores also having lower insulin, HOMA-IR, systolic blood pressure, and hs-CRP, as well as higher HDL-cholesterol compared to those with lower hDPI scores. In contrast, for males the uPDI showed the greatest number of associations with CVD outcomes, but higher scoring participants had higher waist circumference, waist-to-height ratio, insulin, HOMA-IR, and hypertension status, compared to males with lower uPDI scores. This study provides new insights into how less healthy plant-based diets track across adolescence into young adulthood, negatively impacting on cardiometabolic risk factors. Findings suggest that a plant-based diet alone may not suffice for health benefits; the quality of the foods consumed is also essential. Additionally, outcomes from this study highlight the importance of early intervention in adolescence to reduce future risk of CVD.

The present study identified that the hPDI exhibited the greatest number of associations with cardiometabolic health outcomes for females, while the uPDI had the greatest number of associations for males. These included beneficial inverse associations for the hPDI with insulin, HOMA-IR, hs-CRP, and blood pressure. A previous longitudinal analysis of Australian young to mid-aged adults identified that the hPDI was associated with higher insulin sensitivity, and that waist circumference mediated this pathway [54]. Additionally, US longitudinal data from the National Health and Nutrition Examination Survey (NHANES) identified both the PDI and hPDI were associated with lower hs-CRP in mid aged adults, and the Nurses’ Health Study found an increase in hPDI score over time was inversely associated with hs-CRP [55, 56]. While cross sectional data of mid aged adults from Japan, China, the UK and the US reported that the hPDI was associated with lower blood pressure [57], but not the PDI, as per the results in this analysis. Moreover, analysis of three prospective cohort studies from the US reported the hPDI was associated with less weight gain over time, with less significant results found for the PDI [58]. However, there is a lack of research using other measurements such as waist-to-height ratio or percentage fat mass, and a lack of longitudinal evidence using younger age brackets, with most research previously conducted using US data. Furthermore, the greater number of associations, as well as their strength and association directions found for the hPDI and uPDI compared to the PDI in this study also indicate the importance for future plant-based diet research to differentiate by the healthiness of the diet, compared to just differentiating between animal or plant-based foods. As there is higher consumption of discretionary or ultra-processed foods in younger age brackets [59], and an increase in the availability of UPF over recent years [60], this further highlights the need to distinguish between healthy and less healthy plant-based foods when examining diets in younger age groups.

Associations with cardiometabolic health outcomes varied between the sexes in this study. Previous research has identified that sex-specific dietary patterns influence cardiovascular disease risk differently in men and women [17]. A US study using NHANES data found that a “Western” dietary pattern high in animal-sourced foods was positively associated with serum insulin in adults [61], and a cross-sectional analysis identified the metabolic syndrome was associated with higher adherence to an “unhealthy” dietary pattern in men, and to higher adherence to a “healthy” dietary pattern in women [62]. This aligns with the present study which identified, for males, being in the group with higher uPDI scores was positively associated with higher insulin. Females with a higher hPDI score from the present analysis also had associations with insulin, however their remaining associations were related to blood lipids and hs-CRP. A cross-sectional analysis of young Brazilian adults identified a “common Brazilian” dietary pattern was inversely associated with total, LDL and HDL cholesterol in women [63], and a longitudinal analysis of the Cardiovascular Risk in Young Finns Study identified significant inverse associations between the “health-conscious” dietary pattern scores with total and LDL cholesterol, and inflammation in women but not men [64]. Understanding sex and gender differences in CVD prevalence and outcomes is complex [65]. Factors such as female sex hormones have been suggested as an explanation of blood pressure differences between males and females [65–67]. Additionally, metabolic differences such as fat distribution [68, 69] and insulin sensitivity [70, 71], alongside behavioural factors such as smoking [72, 73] and physical activity [74] may also contribute to the observed disparities in cardiovascular health across the sexes [65]. Thus, associations between dietary patterns and cardiometabolic health markers vary for males and females, and understanding these sex-based differences is important for more tailored prevention strategies in cardiometabolic health.

Our study highlights the importance of maintaining a healthy diet over all life stages. Plant-based diet quality trajectory groups were relatively stable over the study period with groups remaining either above or below the average diet quality at all time points. Previous research using the Raine Study showed a less healthy “Western” dietary pattern in adolescence persisted into early adulthood, in particular for males who had greater stability in this pattern over time [75]. Conversely, a Norwegian study which followed participants from 14 to 30 years of age showed fruit and vegetable intake declined from adolescence to early twenties, before increasing to age 30 [76]. A Canadian study which followed participants from 8 to 34 years of age identified increasing adherence to vegetarian-style dietary patterns for both sexes over time and increasing scores for a less healthy “Western” dietary pattern for males only [77]. This suggests that healthy dietary behaviours in childhood and adolescence moderately track into young adulthood. Similarly, an additional Canadian study which followed participants from 11 to 18 years of age found dietary patterns worsened over this time [78]. A US study of 2,524 people followed from 15 to 31 years of age found for both sexes that adherence to the Dietary Approached to Stop Hypertension index decreased from adolescence to early twenties, before improving to the early thirties [79]. Overall, they found that males had worse adherence to this index, and that across early adulthood the sex-based differences for the index increased in scale. However, few studies have included more than three dietary assessments time points [18] and, evidence assessing plant-based diets over this life period is limited. Ultimately, this study in combination with previous evidence highlights the growing recognition of the importance of this transitional life stage, and identifies the importance of addressing poor dietary behaviours in early adolescence, to improve diet quality in young adulthood [80].

This study had several strengths. Firstly, longitudinal data was used, allowing several dietary assessments time points to be tracked in the same group of individuals from adolescence to young adulthood, of which few exist [18]. Additionally, to assess these dietary time points three validated plant-based diet quality indices were used which differentiated between the quality of the foods, rather than if they were solely plant or animal-based [29]. The cardiovascular health outcome data were objectively measured and not self-reported, reducing the risk of bias and measurements were collected a year after their last dietary assessment, avoiding potential reverse causality in the analysis.

There were also several limitations to the analysis. Different FFQs with different foods and beverages were administered over the analysis period. However, previous research has shown these are comparable at analysing diet over time [81]. Dietary assessments were self-reported and thus may have introduced mis-reporting biases. However, including energy misreporting in the main analysis, and conducting a sensitivity analysis that excluded energy mis reporters at any time point mitigated this issue to some extent. Another limitation was the use of multiple testing which can increase the risk of Type I error. However, this was addressed by using appropriate statistical adjustments and interpreting the findings within the exploratory context of the analysis. Lastly, covariates were assessed from the Gen2-14 year follow-up, which means they may have changed over time and potentially influenced the outcomes. It is also important to acknowledge any unmeasured and residual confounding from other dietary, behaviour, or sociodemographic factors.

This study highlights some important considerations for future research and public health promotion. Future research could consider the use of a cardiometabolic risk score which may account for the interplay between various risk factors, which may not be apparent when analysed individually. Additionally, while this analysis was sex-specific, future research including intersex people and separate analysis by gender is needed [82]. Moreover, the Raine Study Gen2 participants were not a nationally representative population, and predominantly represented Caucasians, thus future research with more diverse populations is warranted. Lastly, as the nutrition transition from adolescence into adulthood is affected by a multitude of factors [83], future research should seek to understand the various drivers of adolescent food choices and how this then impacts nutritional intake in adulthood.

## Conclusions

This study identified that plant-based diet quality scores were relatively stable from adolescence into young adulthood, with participants remaining either above or below average diet quality at all time points. Plant-based diet quality trajectory groups, as well as associations with cardiometabolic health outcomes differed for males and females. The hPDI exhibited the greatest number of associations for females, all of which were beneficial for cardiometabolic health, while the uPDI had the greatest number of associations for males, with all resulting in poorer cardiometabolic health. These findings provide new insights into the dietary transition from adolescence to adulthood and highlight the importance of promoting healthy dietary patterns at all life stages. Additionally, this research provides evidence of the need for sex specific research and early interventions in adolescence to reduce future risk of CVD.

## Electronic supplementary material

Below is the link to the electronic supplementary material.

Supplementary Material 1

## Data Availability

No datasets were generated or analysed during the current study.

## Abbreviations

BIC: Bayesian information criterion
CVD: Cardiovascular disease
CSIRO: Commonwealth scientific and industrial research organisation
DQESV2: Quantitative dietary questionnaire for epidemiological studies
FFQ: Food frequency questionnaire
Gen1: Generation 1
Gen2: Generation 2
GRoLTS: Guidelines for reporting on latent trajectory studies
HDL: High-density lipoprotein
Hs-CRP: High-sensitivity C-reactive protein
HOMA-IR: Homeostatic model assessment for insulin resistance
hPDI: Healthy plant-based diet index
IQR: Interquartile range
NHANES: National health and nutrition examination survey
PDI: Plant-based diet index
STROBE-nut: Strengthening the reporting of observational studies in epidemiology – nutritional epidemiology
TC:HDL: Total cholesterol to high-density lipoprotein cholesterol ratio
uPDI: Less healthy plant-based diet index
