# Supplementary material for: Trajectories of plant-based dietary patterns and their sex-specific associations with cardiometabolic health among young Australian adults
Source: Int J Behav Nutr Phys Act. 2025 May 27;22:62. doi: 10.1186/s12966-025-01765-0 (PMC12117903; doi:10.1186/s12966-025-01765-0)
Supplement: Supplementary file 1 — Supplementary Material 1 [file 12966_2025_1765_MOESM1_ESM.docx]

**Title:**

Trajectories of plant-based dietary patterns and their sex-specific associations with cardiometabolic health among young Australian adults

**Corresponding author:**

Laura Marchese, Institute for Physical Activity and Nutrition, School of Exercise and Nutrition Sciences, Deakin University, Melbourne Burwood Campus, 221 Burwood Highway, Victoria 3125, Australia

# Supplementary Material

## Supplementary Table 1: Strengthening the Reporting of Observational studies in Epidemiology – Nutritional Epidemiology (STROBE-nut) reporting guidelines (1)

| **Item** | **Item nr** | **STROBE recommendations** | **Extension for Nutritional Epidemiology studies (STROBE-nut)** | **Reported on page #** |
| --- | --- | --- | --- | --- |
| **Title and**  **abstract** | 1 | (a) Indicate the study’s design with a commonly used term in the title or the abstract.  (b) Provide in the abstract an informative and balanced summary of what was done and what was found. | **nut-1** State the dietary/nutritional assessment method(s) used in the title, abstract, or keywords. | 1-4 |
| **Introduction** |  |  |  | 5-6 |
| Background rationale | 2 | Explain the scientific background and rationale for the investigation being reported. |  | 5-6 |
| Objectives | 3 | State specific objectives, including any pre-specified hypotheses. |  | 6 |
| **Methods** |  |  |  | 6-12 |
| Study design | 4 | Present key elements of study design early in the paper. |  | 6-7 |
| Settings | 5 | Describe the setting, locations, and relevant dates, including periods of recruitment, exposure, follow-up, and data collection. | **nut-5** Describe any characteristics of the study settings that might affect the dietary intake or nutritional status of the participants, if applicable. | 6-10 |
| Participants | 6 | a) Cohort study—Give the eligibility criteria, and the sources and methods of selection of participants. Describe methods of follow-up.  Case-control study—Give the eligibility criteria, and the sources and methods of case ascertainment and control selection. Give the rationale for the choice of cases and controls.  Cross-sectional study—Give the eligibility criteria, and the sources and methods of selection of participants.  (b) Cohort study—For matched studies, give matching criteria and number of exposed and unexposed.  Case-control study—For matched studies, give matching criteria and the number of controls per case. | **nut-6** Report particular dietary, physiological or nutritional characteristics that were considered when selecting the target population. | 10 |
| Variables | 7 | Clearly define all outcomes, exposures, predictors, potential confounders, and effect modifiers. Give diagnostic criteria, if applicable. | **nut-7.1** Clearly define foods, food groups, nutrients, or other food components.  **nut-7.2** When using dietary patterns or indices, describe the methods to obtain them and their nutritional properties. | 7-9  7-9 |
| Data sources - measurements | 8 | For each variable of interest, give sources of data and details of methods of assessment (measurement).Describe comparability of assessment methods if there is more than one group. | **nut-8.1** Describe the dietary assessment method(s), e.g., portion size estimation, number of days and items recorded, how it was developed and administered, and how quality was assured. Report if and how supplement intake was assessed.  **nut-8.2** Describe and justify food composition data used. Explain the procedure to match food composition with consumption data. Describe the use of conversion factors, if applicable.  **nut-8.3** Describe the nutrient requirements, recommendations, or dietary guidelines and the evaluation approach used to compare intake with the dietary reference values, if applicable.  **nut-8.4** When using nutritional biomarkers, additionally use the STROBE Extension for Molecular Epidemiology (STROBE-ME). Report the type of biomarkers used and their usefulness as dietary exposure markers.  **nut-8.5** Describe the assessment of nondietary data (e.g., nutritional status and influencing factors) and timing of the assessment of these variables in relation to dietary assessment.  **nut-8.6** Report on the validity of the dietary or nutritional assessment methods and any internal or external validation used in the study, if applicable. | 7-8  Not applicable  Not applicable  Not applicable  Not applicable  7-8 |
| Bias | 9 | Describe any efforts to address potential sources of bias. | **nut-9** Report how bias in dietary or nutritional assessment was addressed, e.g., misreporting, changes in habits as a result of being measured, or data imputation from other sources | 8 |
| Study Size | 10 | Explain how the study size was arrived at. |  | 12 and supplementary figure |
| Quantitative variables | 11 | Explain how quantitative variables were handled in the analyses. If applicable, describe which groupings were chosen and why. | **nut-11** Explain categorization of dietary/nutritional data (e.g., use of N-tiles and handling of nonconsumers) and the choice of reference category, if applicable. | 10-12 |
| Statistical  Methods | 12 | (a) Describe all statistical methods, including those used to control for confounding  (b) Describe any methods used to examine subgroups and interactions.  (c) Explain how missing data were addressed.  (d) Cohort study—If applicable, explain how loss to follow-up was addressed.  Case-control study—If applicable, explain how matching of cases and controls was addressed.  Cross-sectional study—If applicable, describe analytical methods taking account of sampling strategy.  (e) Describe any sensitivity analyses. | **nut-12.1** Describe any statistical method used to combine dietary or nutritional data, if applicable.  **nut-12.2** Describe and justify the method for energy adjustments, intake modeling, and use of weighting factors, if applicable.  **nut-12.3** Report any adjustments for measurement error, i.e, from a validity or calibration study. | 10-12  8-9, 10-12  12 |
| **Results** |  |  |  | 12-20 |
| Participants | 13 | (a) Report the numbers of individuals at each stage of the study—e.g., numbers potentially eligible, examined for eligibility, confirmed eligible, included in the study, completing follow-up, and analyzed.  (b) Give reasons for non-participation at each stage.  (c) Consider use of a flow diagram. | **nut-13** Report the number of individuals excluded based on missing, incomplete or implausible dietary/nutritional data. | 12 and supplementary figure |
| Descriptive data | 14 | (a) Give characteristics of study participants (e.g., demographic, clinical, social) and information on exposures and potential confounders  (b) Indicate the number of participants with missing data for each variable of interest  (c) Cohort study—Summarize follow-up time (e.g., average and total amount) | **nut-14** Give the distribution of participant characteristics across the exposure variables if applicable. Specify if food consumption of total population or consumers only were used to obtain results. | Tables 1 and 2 |
| Outcome data | 15 | Cohort study—Report numbers of outcome events or summary measures over time.  Case-control study—Report numbers in each exposure category, or summary measures of exposure.  Cross-sectional study—Report numbers of outcome events or summary measures. |  | Tables 1 and 2 |
| Main results | 16 | (a) Give unadjusted estimates and, if applicable, confounder-adjusted estimates and their precision (e.g., 95% confidence interval).  Make clear which confounders were adjusted for and why they were included.  (b) Report category boundaries when continuous variables were categorized.  (c) If relevant, consider translating estimates of relative risk into absolute risk for a meaningful time period. | **nut-16** Specify if nutrient intakes are reported with or without inclusion of dietary supplement intake, if applicable. | Not applicable |
| Other analyses | 17 | Report other analyses done—e.g., analyses of subgroups and interactions and sensitivity analyses. | **nut-17** Report any sensitivity analysis (e.g., exclusion of misreporters or outliers) and data imputation, if applicable. | 20 |
| **Discussion** |  |  |  | 20-25 |
| Key results | 18 | Summarize key results with reference to study objectives. |  | 20-21 |
| Limitation | 19 | Discuss limitations of the study, taking into account sources of potential bias or imprecision. Discuss both direction and magnitude of any potential bias. | **nut-19** Describe the main limitations of the data sources and assessment methods used and implications for the interpretation of the findings. | 24 |
| Interpretation | 20 | Give a cautious overall interpretation of results considering objectives, limitations, multiplicity of analyses, results from similar studies, and other relevant evidence. | **nut-20** Report the nutritional relevance of the findings, given the complexity of diet or nutrition as an exposure. | 23-25 |
| Generalizability | 21 | Discuss the generalizability (external validity) of the study results. |  | 20-25 |
| **Other information** |  |  |  | 25-28 |
| Funding | 22 | Give the source of funding and the role of the funders for the present study and, if applicable, for the original study on which the present article is based. |  | 26-28 |
| *Ethics* |  |  | **nut-22.1** Describe the procedure for consent and study approval from ethics committee(s). | 26 |
| *Supplementary material* |  |  | **nut-22.2** Provide data collection tools and data as online material or explain how they can be accessed. | Separate document or https://rainestudy.org.au/ |

## Supplementary Table 2: Guidelines for Reporting on Latent Trajectory Studies (GRoLTS) Checklist (2)

|  | **Checklist Item** | **Reported?** |
| --- | --- | --- |
| **1** | Is the metric of time used in the statistical model reported? | Yes |
| **2** | Is information presented about the mean and variance of time within a wave? | No |
| **3a.** | Is the missing data mechanism reported? | Yes |
| **3b.** | Is a description provided of what variables are related to attrition/missing data? | Yes (supplementary table 5) |
| **3c.** | Is a description provided of how missing data in the analyses were dealt with? | Yes |
| **4** | Is information about the distribution of the observed variables included? | Yes |
| **5** | Is the software mentioned? | Yes |
| **6a.** | Are alternative specifications of within-class heterogeneity considered (e.g., LGCA vs. LGMM) and clearly documented? If not, was sufficient justification provided as to eliminate certain specifications from consideration? | No |
| **6b.** | Are alternative specifications of the between-class differences in variance-covariance matrix structure considered and clearly documented? If not, was sufficient justification provided as to eliminate certain specifications from consideration? | No (not an option using STATA traj command) |
| **7** | Are alternative shape/functional forms of the trajectories described? | No |
| **8** | If covariates have been used, can analyses still be replicated? | Yes |
| **9** | Is information reported about the number of random start values and final iterations included? | No |
| **10** | Are the model comparison (and selection) tools described from a statistical perspective? | Yes (supplementary table 4) |
| **11** | Are the total number of fitted models reported, including a one-class solution? | Yes |
| **12** | Are the number of cases per class reported for each model (absolute sample size, or proportion)? | Yes |
| **13** | If classification of cases in a trajectory is the goal, is entropy reported? | Yes (supplementary table 4) |
| **14a.** | Is a plot included with the estimated mean trajectories of the final solution? | Yes |
| **14b.** | Are plots included with the estimated mean trajectories for each model? | Yes |
| **14c.** | Is a plot included of the combination of estimated means of the final model and the observed individual trajectories split out for each latent class? | Yes |
| **15** | Are characteristics of the final class solution numerically described (i.e., means, SD/SE, n, CI, etc.)? | Yes |
| **16** | Are the syntax files available (either in the appendix, supplementary materials, or from the authors)? | Yes |

## Supplementary Table 3: Food frequency questionnaire items assigned to the 17 food groups, and their scoring across the three plant-based diet quality indices

|  | **Gen2-14 year follow-up (CSIRO FFQ)** | **Gen2-20 year follow-up (DQESV2 FFQ)** | **Gen2-27 year follow-up (DQESV2 FFQ)** | **PDI** | **hPDI** | **uPDI** |
| --- | --- | --- | --- | --- | --- | --- |
| **Plant Food Groups** | | | | | | |
| **Healthy** | | | | | | |
| Whole grains | Porridge/oatmeal, muesli, bran, wheatgerm. | All Bran, branflakes, multi-grain bread, rye bread, wholemeal bread, muesli, porridge, and Weet Bix. | Wholemeal crackers or dry biscuits, multigrain bread, rye bread, soy and linseed bread, wholemeal bread, toasted muesli, non-toasted muesli, mixed grain cereal, bran cereal, average porridge, Weet-Bix and flakes cereal, and Special K. | Positive scores | Positive scores | Reverse scores |
| Fruits | Orange/citrus fruit, apple/pear, banana, fresh fruit salad, dried fruit (apple/apricot), raisins/currants/sultanas, fruit canned in syrup/stewed, fruit canned in water (low cal), berries in season, melon (rock) in season, peach in season, plum in season, nectarine in season, apricot in season, grapes in season, pineapple in season, extra fruit. | Apples, apricots, bananas, tinned fruit, mango, melon, oranges, peaches, pears, pineapple, and strawberries. | Apples, dried apricots, apricots, bananas, other berries, cherries, dried fruit, tinned fruit, grapes, kiwi fruit, mango, watermelon, rockmelon, oranges and other citrus fruits, peaches, pears, pineapple, plums, and strawberries. | Positive scores | Positive scores | Reverse scores |
| Vegetables | Coleslaw (summer), coleslaw (winter), avocado (in season), carrots (summer), carrots (winter), turnip/swede (summer), turnip/swede (winter), broad beans (summer), broad beans (winter), green peas (summer), green peas (winter), cabbage (summer), cabbage (winter), brussels sprouts (summer), brussels sprouts (winter), silverbeet/spinach (summer), silverbeet/spinach (winter), broccoli (summer), broccoli (winter), cauliflower (summer), cauliflower (winter), pumpkin (summer), pumpkin (winter), sweetcorn (summer), sweetcorn (winter), zucchini (summer), zucchini (winter), fried onion (summer), fried onion (winter), raw onion (summer), raw onion (winter), fresh tomato (summer), fresh tomato (winter), fried tomato (summer), fried tomato (winter), lettuce (summer), lettuce (winter), cucumber (summer), cucumber (winter), celery (summer), celery (winter), capsicum (summer), capsicum (winter), fresh mushrooms (summer), fresh mushrooms (winter), sprouted bean shoots (summer), sprouted bean shoots (winter), fried mixed vegetables (summer), fried mixed vegetables (winter), canned carrots, canned beetroot, canned green peas, canned sweetcorn, canned mushrooms, canned tomatoes in sauce, canned pureed vegetables, zucchini salad, olives, pickled gherkins. | Avocado, bean sprouts, beetroot, broccoli, cabbage, carrots, cauliflower, celery, cucumber, garlic, lettuce (endive or other green salad), mushrooms, onion, peas, peppers (capsicum), pumpkin, spinach, zucchini, and tomatoes. | Asparagus, avocado, beetroot, broccoli, cabbage, carrots, cauliflower, celery, coleslaw, sweetcorn, cucumber, eggplant, figs, garlic, Asian greens, iceberg lettuce, other lettuce, mushrooms, olives, onion, peas, capsicum, pumpkin, spinach and other cooked greens, tomatoes, tinned tomatoes, and zucchini. | Positive scores | Positive scores | Reverse scores |
| Nuts | Fresh peanuts, salted and cooked nuts, other unsalted nuts. | Nuts and peanut butter. | Other nuts and peanuts (or peanut butter). | Positive scores | Positive scores | Reverse scores |
| Legumes | Green beans (summer), green beans (winter), green beans (canned), haricot/lima beans (canned), baked beans, lentils (dried/canned). | Other beans, baked beans, green beans, soybeans (including soy bean curd or tofu), and soya milk. | Green beans, other beans, rice/oat/other milk, baked beans, and soya milk | Positive scores | Positive scores | Reverse scores |
| Tea and coffee | Tea, herbal tea, instant coffee, ground coffee, decaffeinated coffee, coffee substitute. | *Teas, herbal teas, green teas, instant coffees, ground coffees | Coffee, coffee substitute, tea, and herbal tea. | Positive scores | Positive scores | Reverse scores |
| **Less Healthy** | | | | | | |
| Fruit juices | Vegetable juice, pure fruit juice, fruit drink. | Fruit juice | Orange juice and other fruit juice. | Positive scores | Reverse scores | Positive scores |
| Refined grains | Other pasta, crumpet/muffin, salted biscuits, plain biscuits, sliced bread, bread roll, fruit loaf/currant bread, sweet bun/doughnut, crispbread/cracker, other breakfast cereal, boiled rice, instant noodles, muesli bar/health bar. | High fibre white bread, dry biscuits (including crackers and crispbread), white bread, cornflakes, pasta, and rice. | Rice, gluten-free bread, white bread, average cereal, cornflakes, Nutrigrain, sugary cereal, pasta or noodles, rice cereal, and high fibre bread. | Positive scores | Reverse scores | Positive scores |
| Potatoes | Mashed potato (summer), mashed potato (winter), boiled potato (summer), boiled potato (winter), roasted potato (summer), roasted potato (winter), hot chips (summer), hot chips (winter), potato gems (summer), potato gems (winter), canned potato, packet potato, potato salad, crisps/twisties. | Crisps, potatoes cooked without fat, and roasted or fried potatoes (including hot chips). | Potato, sweet potato, crisps and corn chips, and chips or potatoes cooked in or served with fat. | Positive scores | Reverse scores | Positive scores |
| Sugar sweetened beverages | Cordial, coke, soft drink, low-calorie soft drink. | *Fizzy drinks, diet drinks, energy drinks, diet energy drinks | Diet soft drinks and soft drinks. | Positive scores | Reverse scores | Positive scores |
| Sweets and desserts | Fancy biscuits, light or rich cake, fruit pie or pastry, milk pudding, steamed pudding, chocolate, chocolate-covered bars (e.g., Mars), individual lollies/toffees, packet lollies (Lifesavers), honey/jam/marmalade, ice blocks (summer), ice blocks (winter), custard, croissant, Vitari cone (summer), Vitari cone (winter). | Sweet biscuits, cakes, chocolate, and jam. | Other biscuits, sweet biscuits, cake, chocolate, other confectionery, and jam. | Positive scores | Reverse scores | Positive scores |
| **Animal Food Groups** | | | | | | |
| Animal fat | Fat spreads | Monounsaturated margarine, margarine, butter, polyunsaturated margarine, and butter and margarine blends. | Olive oil spread, butter, butter and margarine blends, butter on cooked vegetables, margarine on cooked vegetables, canola margarine, cholesterol-lowering margarine, olive oil margarine, and polyunsaturated margarine. | Reverse scores | Reverse scores | Reverse scores |
| Dairy | Added milk, plain milk, cheese, cottage cheese, cream, yoghurt, flavoured milk, milkshake/thick shake, ice cream (tub - summer), ice cream (tub - winter), ice cream desserts (summer), ice cream desserts (winter), ice cream (cone - summer), ice cream (cone - winter). | Low fat cheese, soft cheese, firm cheese, hard cheese, cream cheese, ricotta or cottage cheese, flavoured milk drink, full cream milk, reduced fat milk, skim milk, yoghurt, cheese, and ice cream. | Other cheese, ricotta and cottage cheese, cream or sour cream, flavoured milk, full cream milk, reduced fat milk, skim milk, and yoghurt. | Reverse scores | Reverse scores | Reverse scores |
| Egg | Fried egg, boiled egg, omelette/scrambled eggs. | Eggs | Eggs | Reverse scores | Reverse scores | Reverse scores |
| Fish or seafood | Fish fried, fish without batter, canned fish, fish fingers, seafood. | Fried fish, fish, and tinned fish. | Fried fish, steamed fish, tinned fish, and other seafood. | Reverse scores | Reverse scores | Reverse scores |
| Meat | Steak, pork chop, lamb chop, roast pork, roast beef/veal, roast lamb, sausages, hamburger patty (no bun), frankfurters/saveloys, bacon, ham, fritz/devon, salami/mettwurst, pâté/liver paste, liver, kidney, brains, spicy mince (e.g., pasta sauce), mince meat (eaten as such), roast chicken, boiled chicken, fried/BBQ chicken, chicken nuggets. | Bacon, beef, corned beef (including luncheon meats or salami), chicken, ham, hamburger, lamb, pork, sausages, and veal. | Bacon, beef and veal, chicken, lamb, pork, processed meat, and sausages. | Reverse scores | Reverse scores | Reverse scores |
| Miscellaneous animal-based foods | Mornay dishes, pureed meat dishes, hamburger with bun, frozen pizza, homemade or takeaway pizza, sausage roll, shop meat pie, home meat pie, pastie, schnitzel, stew/casserole, curry/goulash, Chinese meat & vegetables, savory pies, mince meat dishes, polyunsaturated mayonnaise/salad cream, regular mayonnaise/salad cream, fried rice. | Meat pies (including pastries, quiche, and other savory pastries) and pizza. | Pizza, mayonnaise, quiche and pastries with cheese, and meat pies and pastries with meat. | Reverse scores | Reverse scores | Reverse scores |

*sourced from semiquantitative beverage questionnaire

## Supplementary Table 4: Table of competing models from the group based trajectory modelling

| **Sex** | **Index** | **Model** | **BIC (N=557)** | **BIC (N=201)** | **Entropy** | **Smallest group %** |
| --- | --- | --- | --- | --- | --- | --- |
| **Females** | **PDI** | **(2 2)** | -803.57 | -799.49 | 0.580 | 20.46 |
| Females | PDI | (2 2 2) | -814.40 | -808.29 | 0.577 | 10.92 |
| Females | PDI | (2 2 2 2) | -823.84 | -815.69 | 0.484 | 14.28 |
| Females | PDI | (2 2 2 2 2) | -835.49 | -825.30 | 0.505 | 1.88 |
| **Females** | **hPDI** | **(2 2)** | -795.23 | -791.16 | 0.467 | 46.19 |
| Females | hPDI | (2 2 2) | -798.07 | -791.95 | 0.563 | 27.88 |
| Females | hPDI | (2 2 2 2) | -806.80 | -798.64 | 0.641 | 4.05 |
| Females | hPDI | (2 2 2 2 2) | -818.61 | -808.42 | 0.604 | 3.67 |
| **Females** | **uPDI** | **(2 2)** | -787.55 | -783.48 | 0.538 | 47.98 |
| Females | uPDI | (2 2 2) | -796.53 | -790.41 | 0.542 | 13.57 |
| Females | uPDI | (2 2 2 2) | -802.78 | -794.63 | 0.610 | 12.02 |
| Females | uPDI | (2 2 2 2 2) | -810.71 | -800.52 | 0.654 | 3.70 |
|  |  |  | **BIC (N=608)** | **BIC (N=216)** | **Entropy** | **Smallest group %** |
| **Males** | **PDI** | **(2 2)** | -874.91 | -870.77 | 0.430 | 36.44 |
| Males | PDI | (2 2 2) | -881.56 | -875.35 | 0.617 | 5.33 |
| Males | PDI | (2 2 2 2) | -890.94 | -882.66 | 0.675 | 4.57 |
| Males | PDI | (2 2 2 2 2) | -902.53 | -892.18 | 0.586 | 4.84 |
| **Males** | **hPDI** | **(2 2)** | -863.35 | -859.21 | 0.523 | 48.63 |
| Males | hPDI | (2 2 2) | -867.39 | -861.18 | 0.711 | 1.34 |
| Males | hPDI | (2 2 2 2) | -874.62 | -866.34 | 0.779 | 1.35 |
| Males | hPDI | (2 2 2 2 2) | -885.66 | -875.31 | 0.665 | 1.36 |
| **Males** | **uPDI** | **(2 2)** | -859.24 | -855.1 | 0.521 | 43.98 |
| Males | uPDI | (2 2 2) | -867.24 | -861.03 | 0.559 | 9.62 |
| Males | uPDI | (2 2 2 2) | -873.33 | -865.05 | 0.582 | 8.64 |
| Males | uPDI | (2 2 2 2 2) | -878.5 | -868.15 | 0.664 | 3.13 |

PDI: plant-based diet index, hPDI: healthy plant-based diet index, uPDI: less healthy plant-based diet index, BIC: Bayesian information criterion

## Supplementary Figure 1: Directed acyclic graph used to identify the covariate selection for the statistical analysis


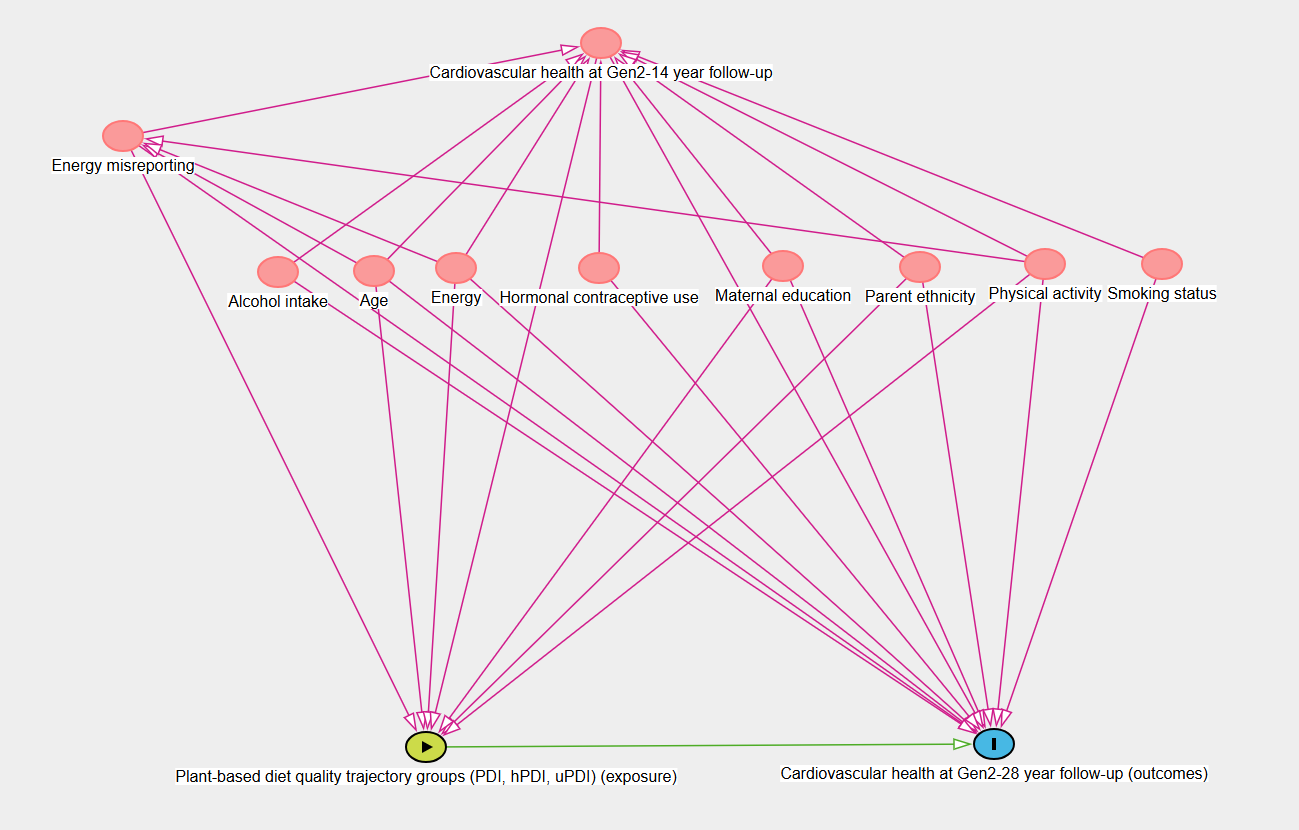


## Supplementary Figure 2: Flow diagram of participants included in the analysis 
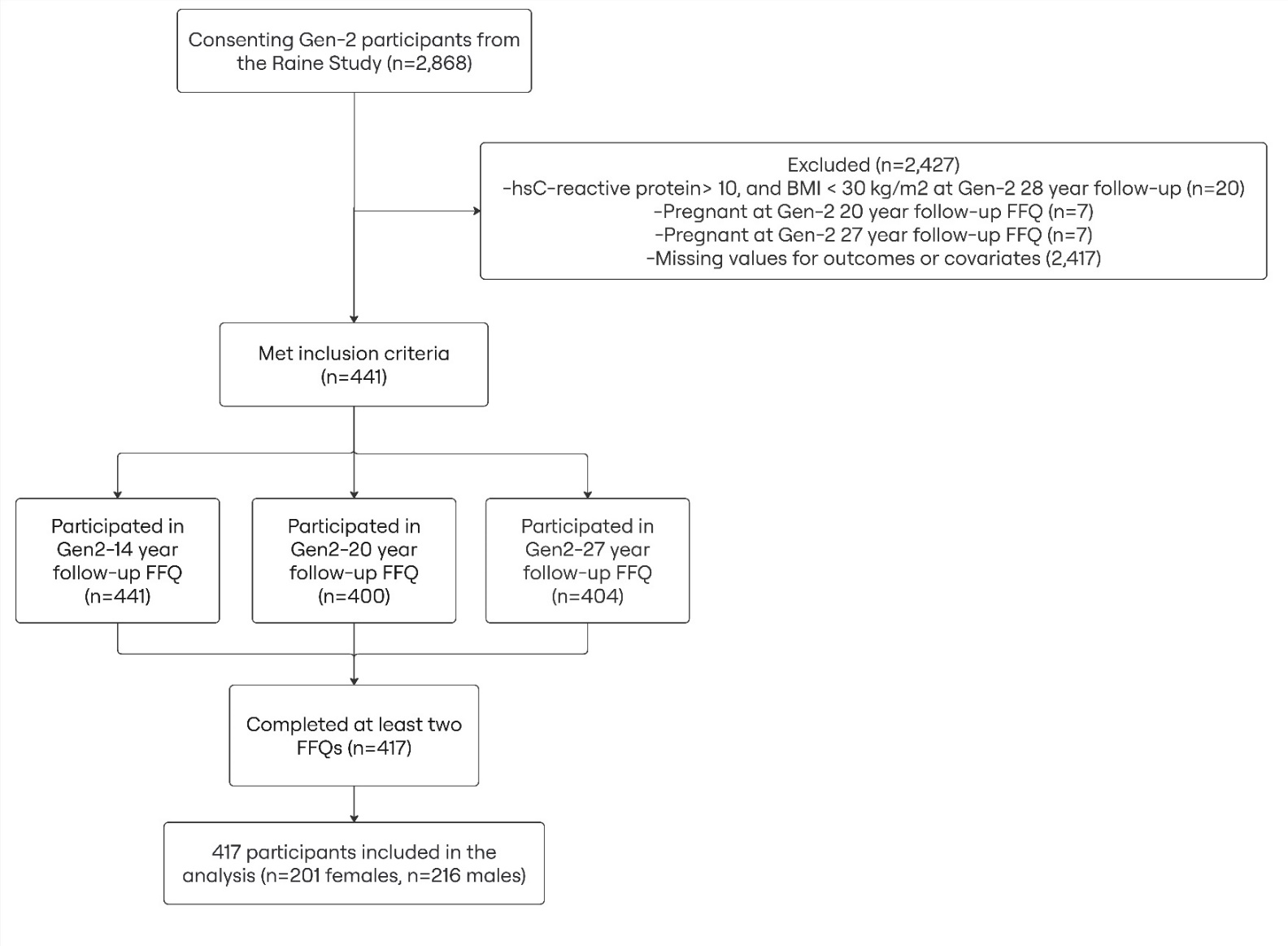


## Supplementary Table 5: Characteristics of the Raine Study Gen2-14 year follow-up participants included and excluded in the analysis (n=2,868)

| **Characteristic** | **Included** | **Excluded** | **P-value** |
| --- | --- | --- | --- |
| N | 417 (14.54) | 2,451 (85.46) |  |
| Female | 201 (48.20) | 1,212 (49.45) | 0.638 |
| Age (years), mean SD | 14.08 (±0.19) | 14.14 (±0.22) | <0.001 |
| Maternal education ^a^ |  |  | <0.001 |
| Tertiary education | 172 (41.25) | 467 (29.92) |  |
| No Tertiary education | 245 (58.75) | 1,094 (70.08) |  |
| Ethnicity ^a^ |  |  | 0.002 |
| Both parents Caucasian | 366 (87.77) | 2,002 (81.68) |  |
| Other | 51 (12.23) | 449 (18.32) |  |
| Smoking status |  |  | 0.002 |
| Smoker | 73 (17.51) | 340 (24.73) |  |
| Non-smoker | 344 (82.49) | 1,035 (75.27) |  |
| Alcohol ^b^ |  |  | 0.913 |
| No | 170 (40.77) | 474 (41.07) |  |
| Yes | 247 (59.23) | 680 (58.93) |  |
| Contraceptive use (females only) |  |  | 0.030 |
| No | 195 (97.01) | 1,196 (98.68) |  |
| Yes | (6 (2.99) | 16 (1.32) |  |
| Physical activity ^c^ |  |  | 0.059 |
| Low active | 171 (41.01) | 547 (46.36) |  |
| Active | 246 (58.99) | 633 (53.64) |  |
| Energy intake (kj), median (IQR) | 9184 (7442-11159) | 9386 (7468-11481) | 0.2133 |
| PDI score, mean (±SD) | 49.21 (±5.77) | 49.35 (±6.08)^d^ | 0.6838 |
| hPDI score, mean (±SD) | 49.36 (±8.07) | 49.46 (±7.93)^d^ | 0.8265 |
| uPDI score, mean (±SD) | 52.83 (±7.31) | 52.74 (±7.96)^d^ | 0.8384 |
| Diet misreporting |  |  | 0.449 |
| Valid reporting | 378 (90.65) | 885 (89.30) |  |
| Underreporting | 39 (9.35) | 106 (10.70) |  |

All values are n (%) unless otherwise specified. SD: standard deviation, IQR: interquartile range, PDI: plant-based diet index, hPDI: healthy plant-based diet index, uPDI: less healthy plant-based diet index, ^a^ Collected at Gen1-8 year follow-up, ^b^ alcohol in past 12 months, ^c^ physical activity completed inside and outside of school hours, ^d^ n=1,196.

## Supplementary Table 6: Summary of the plant-based diet quality index sub-groups scores across the food frequency questionnaires for females and males

|  | **PDI** | | | | | | **hPDI** | | | | | | **uPDI** | | | | | |
| --- | --- | --- | --- | --- | --- | --- | --- | --- | --- | --- | --- | --- | --- | --- | --- | --- | --- | --- |
|  | **Gen2-14 year follow-up (CSIRO FFQ)** | | **Gen2-20 year follow-up (DQESV2 FFQ)** | | **Gen2-27 year follow-up (DQESV2 FFQ)** | | **Gen2-14 year follow-up (CSIRO FFQ)** | | **Gen2-20 year follow-up (DQESV2 FFQ)** | | **Gen2-27 year follow-up (DQESV2 FFQ)** | | **Gen2-14 year follow-up (CSIRO FFQ)** | | **Gen2-20 year follow-up (DQESV2 FFQ)** | | **Gen2-27 year follow-up (DQESV2 FFQ)** | |
|  | Mean (±SD) | Min, Max | Mean (±SD) | Min, Max | Mean (±SD) | Min, Max | Mean (±SD) | Min, Max | Mean (±SD) | Min, Max | Mean (±SD) | Min, Max | Mean (±SD) | Min, Max | Mean (±SD) | Min, Max | Mean (±SD) | Min, Max |
| **Females** | | | | | | | | | | | | | | | | | | |
| **n** | 201 | | 180 | | 176 | | 201 | | 180 | | 176 | | 201 | | 180 | | 176 | |
| **Healthy Plant Food Groups** | 16.23 (±4.73) | 6, 29 | 17.72 (±5.09) | 8, 29 | 17.88 (±5.24) | 7, 30 | 16.23 (±4.73) | 6, 29 | 17.72 (±5.09) | 8, 29 | 17.88 (±5.24) | 7, 30 | 19.77 (±4.73) | 7, 30 | 18.28 (±5.09) | 7, 28 | 18.11 (5.24) | 6, 29 |
| **Less Healthy Plant Food Groups** | 14.86 (±4.00) | 6, 24 | 14.51 (±4.16) | 6, 25 | 14.10 (±4.20) | 5, 24 | 15.14 (±4.00) | 6, 24 | 15.49 (±4.16) | 5, 24 | 15.90 (±4.20) | 6, 25 | 14.86 (±4.00) | 6, 24 | 14.51 (±4.16) | 6, 25 | 14.10 (±4.20) | 5, 24 |
| **Animal Food Groups** | 18.14 (±4.61) | 7, 29 | 19.03 (±4.40) | 7, 28 | 18.68 (±4.58) | 9, 30 | 18.14 (±4.61) | 7, 29 | 19.03 (±4.40) | 7, 28 | 18.68 (±4.58) | 9, 30 | 18.14 (±4.61) | 7, 29 | 19.03 (±4.40) | 7, 28 | 18.68 (±4.58) | 9, 30 |
| **Totals** | 49.23 (±5.28) | 36, 67 | 51.26 (±5.87) | 33, 66 | 50.67 (±6.67) | 36, 71 | 49.51 (±7.68) | 29, 67 | 52.24 (±8.42) | 33, 72 | 52.47 (±8.27) | 32, 78 | 52.77 (7.45) | 31, 68 | 51.83 (±7.15) | 35, 72 | 50.90 (±7.78) | 32, 69 |
| **Males** | | | | | | | | | | | | | | | | | | |
| **n** | 216 | | 192 | | 201 | | 216 | | 192 | | 201 | | 216 | | 192 | | 201 | |
| **Healthy Plant Food Groups** | 16.14 (±5.04) | 6, 28 | 17.62 (±5.17) | 7, 29 | 17.92 (±4.91) | 8, 29 | 16.14 (±5.04) | 6, 28 | 17.62 (±5.17) | 7, 29 | 17.92 (±4.91) | 8, 29 | 19.86 (±5.04) | 8, 30 | 18.38 (±5.17) | 7, 29 | 18.08 (±4.91) | 7, 28 |
| **Less Healthy Plant Food Groups** | 14.86 (±3.95) | 6, 23 | 14.58 (±4.15) | 5, 25 | 14.49 (±3.86) | 5, 24 | 15.14 (±3.95) | 7, 24 | 15.42 (±4.15) | 5, 25 | 15.51 (±3.86) | 6, 25 | 14.86 (±3.95) | 6, 23 | 14.58 (±4.15) | 5, 25 | 14.49 (±3.86) | 5, 24 |
| **Animal Food Groups** | 18.16 (±4.09) | 7, 27 | 18.80 (±4.61) | 8, 29 | 18.84 (±4.68) | 7, 30 | 18.16 (±4.09) | 7, 27 | 18.80 (±4.61) | 8, 29 | 18.84 (±4.68) | 7, 30 | 18.16 (±4.09) | 7, 27 | 18.80 (±4.61) | 8, 29 | 18.84 (±4.68) | 7, 30 |
| **Totals** | 49.15 (±6.14) | 33, 63 | 50.99 (±6.75) | 35, 65 | 51.24 (±6.15) | 35, 72 | 49.44 (±7.99) | 29, 71 | 51.84 (±7.90) | 32, 78 | 52.26 (±7.52) | 33, 74 | 52.88 (±7.18) | 36, 69 | 51.76 (±6.91) | 30, 75 | 51.41 (±7.91) | 31, 70 |

PDI: plant-based diet index, hPDI: healthy plant-based diet index, uPDI: less healthy plant-based diet index, CSIRO: Commonwealth Scientific and Industrial Research Organisation, FFQ: food frequency questionnaire, DQESV2: Dietary Questionnaire for Epidemiological Studies, SD: standard deviation

## Supplementary Table 7: Unadjusted associations between female plant-based diet quality score trajectory groups and markers of cardiometabolic health at the Gen2-28 year follow-up (n=201)

|  | **PDI** | | | **hPDI** | | | **uPDI** | | |
| --- | --- | --- | --- | --- | --- | --- | --- | --- | --- |
|  | **Group 1**  **Low** | **Group 2**  **High** | **P value** | **Group 1**  **Low** | **Group 2**  **High** | **P value** | **Group 1**  **Low** | **Group 2**  **High** | **P value** |
| Waist circumference | Reference | 0.20 (-5.36, 2.75) | 0.944 | Reference | -6.03 (-10.12, -1.93) | **0.004*** | Reference | 5.05 (0.94, 9.16) | **0.016*** |
| Waist-to-height ratio | Reference | 0.00 (-0.03, 0.03) | 0.975 | Reference | -0.04 (-0.06, -0.01) | **0.004*** | Reference | 0.03 (0.01, 0.06) | **0.015*** |
| Total cholesterol | Reference | 0.04 (-0.27, 0.36) | 0.795 | Reference | 0.35 (0.12, 0.59) | **0.003*** | Reference | 0.07 (-0.16, 0.31) | 0.540 |
| HDL-cholesterol | Reference | 0 .03 (-0.13, 0.19) | 0.717 | Reference | 0.19 (0.08, 0.31) | **0.001*** | Reference | -0.09 (-0.20, 0.03) | 0.142 |
| Triglycerides | Reference | 0.16 (-0.04, 0.36) | 0.122 | Reference | 0.09 (-0.07, 0.24) | 0.260 | Reference | 0.24 (0.09, 0.39) | **0.002*** |
| Non-HDL-C | Reference | 0.01 (-0.29, 0.31) | 0.933 | Reference | 0.16 (-0.06, 0.39) | 0.156 | Reference | 0.16 (-0.06, 0.39) | 0.160 |
| TC:HDL ratio | Reference | -0.03 (-0.35, 0.28) | 0.837 | Reference | -0.06 (-0.30, 0.17) | 0.605 | Reference | 0.24 (0.00, 0.47) | **0.048*** |
| Glucose | Reference | 0.18 (-0.04, 0.40) | 0.111 | Reference | -0.14 (-0.30, 0.03) | 0.103 | Reference | -0.10 (-0.27, 0.06) | 0.227 |
| Insulin | Reference | 0.47 (-0.96, 1.90) | 0.515 | Reference | -1.26 (-2.33, -0.20) | **0.020*** | Reference | 1.11 (0.05, 2.18) | **0.040*** |
| HOMA-IR | Reference | 0.15 (-0.18, 0.47) | 0.383 | Reference | -0.29 (-0.53, -0.04) | **0.023*** | Reference | 0.22 (-0.22, 0.47) | 0.074 |
| Systolic blood pressure | Reference | 0.05 (-3.69, 3.78) | 0.980 | Reference | -0.69 (-3.50, 2.00) | 0.627 | Reference | 0.81 (-1.99, 3.61) | 0.570 |
| Diastolic blood pressure | Reference | -0.84 (-3.49, 1.81) | 0.534 | Reference | -0.63 (-2.62, 1.36) | 0.533 | Reference | 0.85 (-1.13, 2.84) | 0.398 |
| hs-CRP | Reference | -1.05 (-2.72, 0.62) | 0.218 | Reference | -1.68 (-2.92, -0.45) | **0.008*** | Reference | 1.54 (0.30, 2.78) | **0.015*** |
| Combined pre- hypertension/hypertension status^1^ | Reference | 0.98 (0.11-8.68) | 0.987 | Reference | 0.58 (0.10, 3.26) | 0.539 | Reference | 0.88 (0.18, 4.54) | 0.892 |

All values are β coefficients and 95% CI unless otherwise specified. ^1^ OR and 95% CI, PDI: plant-based diet index, hPDI: healthy plant-based diet index, uPDI: less healthy plant-based diet index, * indicates results p<0.05.

## Supplementary Table 8: Unadjusted associations between male plant-based diet quality score trajectory groups and markers of cardiometabolic health at the Gen2-28 year follow-up

|  | **PDI** | | | **hPDI** | | | **uPDI** | | |
| --- | --- | --- | --- | --- | --- | --- | --- | --- | --- |
|  | **Group 1**  **Low** | **Group 2**  **High** | **P value** | **Group 1**  **Low** | **Group 2**  **High** | **P value** | **Group 1**  **Low** | **Group 2**  **Medium** | **P value** |
| Waist circumference | Reference | -1.27 (-4.43, 1.89) | 0.430 | Reference | -1.58 (-4.60, 1.43) | 0.302 | Reference | 1.84 (-1.20, 4.89) | 0.235 |
| Waist-to-height ratio | Reference | -0.01 (-0.03, 0.01) | 0.198 | Reference | -0.01 (-0.02, 0.01) | 0.341 | Reference | 0.02 (-0.00, 0.03) | 0.063 |
| Total cholesterol | Reference | -0.08 (-0.34, 0.18) | 0.562 | Reference | 0.22 (-0.03, 0.47) | 0.079 | Reference | -0.06 (-0.31, 0.19) | 0.619 |
| HDL-cholesterol | Reference | 0.02 (-0.07, 0.10) | 0.736 | Reference | 0.07 (-0.01, 0.15) | 0.106 | Reference | -0.07 (-0.15, 0.02) | 0.123 |
| Triglycerides | Reference | -0.00 (-0.19, 0.18) | 0.961 | Reference | -0.01 (-0.19, 0.16) | 0.892 | Reference | 0.04 (-0.13, 0.22) | 0.646 |
| Non-HDL-C | Reference | -0.09 (-0.36, 0.18) | 0.510 | Reference | 0.15 (-0.11, 0.41) | 0.252 | Reference | 0.00 (-0.26, 0.27) | 0.983 |
| TC:HDL ratio | Reference | -0.21 (-0.58, 0.15) | 0.256 | Reference | -0.10 (-0.45, 0.25) | 0.575 | Reference | 0.21 (-0.14, 0.57) | 0.240 |
| Glucose | Reference | 0.15 (-0.17, 0.47) | 0.369 | Reference | 0.04 (-0.35, 0.37) | 0.799 | Reference | 0.16 (-0.15, 0.47) | 0.299 |
| Insulin | Reference | -0.53 (-1.84, 0.78) | 0.423 | Reference | 0.19 (-1.06, 1.45) | 0.760 | Reference | 1.56 (0.31, 2.81) | **0.015*** |
| HOMA-IR | Reference | -0.11 (-0.41, 0.19) | 0.460 | Reference | 0.06 (-0.23, 0.34) | 0.696 | Reference | 0.35 (0.07, 0.64) | **0.015*** |
| Systolic blood pressure | Reference | -0.01 (-3.11, 3.09) | 0.995 | Reference | 1.05 (-1.91, 4.01) | 0.484 | Reference | -1.43 (-4.42, 1.55) | 0.344 |
| Diastolic blood pressure | Reference | 1.52 (-0.55, 3.59) | 0.148 | Reference | -0.61 (-2.59, 1.37) | 0.544 | Reference | 0.02 (-1.98, 2.03) | 0.981 |
| hs-CRP | Reference | 0.23 (-0.49, 0.95) | 0.532 | Reference | -0.26 (-0.95, 0.43) | 0.455 | Reference | 0.41 (-0.28, 1.10) | 0.246 |
| Combined pre- hypertension/hypertension status^1^ | Reference | 1.09 (0.26, 4.48) | 0.905 | Reference | 0.79 (0.21, 3.03) | 0.734 | Reference | 2.76 (0.67, 11.33) | 0.159 |

All values are β coefficients and 95% CI unless otherwise specified. ^1^ OR and 95% CI, PDI: plant-based diet index, hPDI: healthy plant-based diet index, uPDI: less healthy plant-based diet index, * indicates results p<0.05.

## Supplementary Table 9: Associations between female plant-based diet quality score trajectory groups and markers of cardiometabolic health at the Gen2-28 year follow-up from the sensitivity analysis (n=76)

|  | **PDI** | | | **hPDI** | | | **uPDI** | | |
| --- | --- | --- | --- | --- | --- | --- | --- | --- | --- |
|  | **Group 1**  **Low** | **Group 2**  **High** | **P value** | **Group 1**  **Low** | **Group 2**  **High** | **P value** | **Group 1**  **Low** | **Group 2**  **High** | **P value** |
| Waist circumference | Reference | 0.58 (-3.90, 5.05) | 0.798 | Reference | -1.94 (-6.73, 2.85) | 0.421 | Reference | 5.07 (0.14, 9.99) | **0.044*** |
| Waist-to-height ratio | Reference | -0.00 (-0.03, 0.03) | 0.984 | Reference | -0.01 (-0.04, 0.02) | 0.347 | Reference | 0.03 (0.00, 0.06) | **0.033*** |
| Total cholesterol | Reference | 0.10 (-0.20, 0.40) | 0.519 | Reference | 0.01 (-0.31, 0.33) | 0.945 | Reference | 0.44 (0.11, 0.77) | **0.011*** |
| HDL-cholesterol | Reference | 0.07 (-0.07, 0.22) | 0.338 | Reference | -0.03 (-0.19, 0.12) | 0.678 | Reference | -0.06 (-0.22, 0.10) | 0.469 |
| Triglycerides | Reference | 0.06 (-0.15, 0.26) | 0.586 | Reference | -0.03 (-0.24, 0.19) | 0.808 | Reference | 0.29 (0.06, 0.51) | **0.012*** |
| Non-HDL-C | Reference | 0.04 (-0.24, 0.31) | 0.798 | Reference | 0.05 (-0.24, 0.34) | 0.748 | Reference | 0.49 (0.20, 0.78) | **0.001*** |
| TC:HDL ratio | Reference | -0.03 (-0.32, 0.27) | 0.854 | Reference | 0.09 (-0.22, 0.40) | 0.564 | Reference | 0.39 (0.08, 0.71) | **0.015*** |
| Glucose | Reference | 0.11 (-0.05, 0.26) | 0.172 | Reference | -0.02 (-0.19, 0.14) | 0.764 | Reference | -0.07 (-0.25, 0.10) | 0.411 |
| Insulin | Reference | -0.28 (-1.49, 0.94) | 0.651 | Reference | -0.09 (-1.42, 1.23) | 0.891 | Reference | 0.74 (-0.62, 2.10) | 0.283 |
| HOMA-IR | Reference | -0.03 (-0.28, 0.22) | 0.803 | Reference | -0.05 (-0.29, 0.26) | 0.922 | Reference | 0.12 (-0.16, 0.41) | 0.392 |
| Systolic blood pressure | Reference | -0.90 (-4.86, 3.07) | 0.654 | Reference | 031 (-3.95, 4.56) | 0.886 | Reference | 2.14 (-2.33, 6.62) | 0.343 |
| Diastolic blood pressure | Reference | -0.70 (-3.88, 2.48) | 0.662 | Reference | 0.69 (-2.75, 4.13) | 0.691 | Reference | 0.77 (-2.85, 4.40) | 0.673 |
| hs-CRP | Reference | -1.17 (-3.11, 0.77) | 0.234 | Reference | -1.73 (-3.82, 0.35) | 0.102 | Reference | 1.99 (-0.17, 4.16) | 0.071 |
| Combined pre- hypertension/hypertension status (n=20) ^1^ | Reference | 0.70 (0.04-14.05) | 0.818 | Insufficient sample size to run test | | | Reference | 1.32 (0.06-27.57) | 0.859 |

All values are β coefficients and 95% CI unless otherwise specified. ^1^ OR and 95% CI, PDI: plant-based diet index, hPDI: healthy plant-based diet index, uPDI: less healthy plant-based diet index, adjusted for ethnicity, maternal education, smoking status, alcohol intake over the past 12 months, hormonal contraceptive use, and physical activity, and energy intake, * indicates results p<0.05.

## Supplementary Table 10: Associations between male plant-based diet quality score trajectory groups and markers of cardiometabolic health at the Gen2-28 year follow-up from the sensitivity analysis (n=89)

|  | **PDI** | | | **hPDI** | | | **uPDI** | | |
| --- | --- | --- | --- | --- | --- | --- | --- | --- | --- |
|  | **Group 1**  **Low** | **Group 2**  **High** | **P value** | **Group 1**  **Low** | **Group 2**  **High** | **P value** | **Group 1**  **Low** | **Group 2**  **Medium** | **P value** |
| Waist circumference | Reference | -2.63 (-7.15, 1.89) | 0.250 | Reference | -3.08 (-7.06, 0.94) | 0.131 | Reference | 1.51 (-2.07, 5.09) | 0.403 |
| Waist-to-height ratio | Reference | -0.01 (-0.04, 0.01) | 0.336 | Reference | -0.01 (-0.03, 0.01) | 0.294 | Reference | 0.01 (-0.01, 0.03) | 0.147 |
| Total cholesterol | Reference | -0.16 (-0.60, 0.27) | 0.465 | Reference | 0.02 (-0.34, 0.41) | 0.935 | Reference | 0.06 (-0.28, 0.40) | 0.737 |
| HDL-cholesterol | Reference | 0.01 (-0.13, 0.14) | 0.924 | Reference | 0.08 (-0.04, 0.19) | 0.194 | Reference | -0.01 (-0.11, 0.09) | 0.835 |
| Triglycerides | Reference | -0.12 (-0.53, 0.28) | 0.549 | Reference | -0.12 (-0.49, 0.25) | 0.523 | Reference | 0.03 (-0.29, 0.35) | 0.852 |
| Non-HDL-C | Reference | -0.18 (-0.60, 0.25) | 0.409 | Reference | -0.08 (-0.46, 0.30) | 0.666 | Reference | 0.06 (-0.27, 0.40) | 0.713 |
| TC:HDL ratio | Reference | -0.17 (-0.38, 0.33) | 0.496 | Reference | -0.23 (-0.68, 0.22) | 0.313 | Reference | 0.05 (-0.35, 0.45) | 0.795 |
| Glucose | Reference | 0.15 (-0.09, 0.38) | 0.212 | Reference | 0.05 (-0.16, 0.26) | 0.609 | Reference | -0.16 (-0.34, 0.02) | 0.083 |
| Insulin | Reference | 0.40 (-0.97, 1.69) | 0.591 | Reference | -0.24 (-1.42, 0.93) | 0.681 | Reference | 0.19 (-0.86, 1.25) | 0.717 |
| HOMA-IR | Reference | 0.09 (-0.21, 0.40) | 0.546 | Reference | -0.04 (-0.31, 0.23) | 0.761 | Reference | 0.01 (-0.23, 0.26) | 0.905 |
| Systolic blood pressure | Reference | 0.44 (-4.45, 5.32) | 0.860 | Reference | -2.07 (-6.41, 2.27) | 0.345 | Reference | 1.16 (-2.68, 5.01) | 0.549 |
| Diastolic blood pressure | Reference | -1.91 (-5.74, 1.92) | 0.325 | Reference | -0.94 (-4.33, 2.45) | 0.581 | Reference | 1.53 (-1.45, 4.51) | 0.311 |
| hs-CRP | Reference | -0.65 (-1.60, 0.31) | 0.180 | Reference | -0.48 (-1.34, 0.39) | 0.277 | Reference | 0.19 (-0.57, 0.95) | 0.617 |
| Combined pre- hypertension/hypertension status (n=78) ^1^ | Reference | 0.42 (0.04, 5.04) | 0.495 | Insufficient sample size to run test | | | | | |

All values are β coefficients and 95% CI unless otherwise specified. ^1^ OR and 95% CI, PDI: plant-based diet index, hPDI: healthy plant-based diet index, uPDI: less healthy plant-based diet index, adjusted for ethnicity, maternal education, smoking status, alcohol intake over the past 12 months, and physical activity, and energy intake.

## Supplementary Table 11: Associations between female plant-based diet quality score trajectory groups and markers of cardiometabolic health at the Gen2-28 year follow-up from the multiple imputation sensitivity analysis (n=248) (number of imputations=20)

|  | **PDI** | | | **hPDI** | | | **uPDI** | | |
| --- | --- | --- | --- | --- | --- | --- | --- | --- | --- |
|  | **Group 1**  **Low** | **Group 2**  **High** | **P value** | **Group 1**  **Low** | **Group 2**  **High** | **P value** | **Group 1**  **Low** | **Group 2**  **High** | **P value** |
| Waist circumference | Reference | -0.14 (-3.27, 3.00) | 0.932 | Reference | -2.37 (-5.71, 0.97) | 0.164 | Reference | 2.78 (-0.25, 5.81) | 0.072 |
| Waist-to-height ratio | Reference | -0.00 (-0.02, 0.02) | 0.798 | Reference | -0.02 (-0.04, 0.00) | 0.121 | Reference | 0.02 (0.00, 0.04) | **0.042*** |
| Total cholesterol | Reference | -0.05 (-0.25, 0.15) | 0.610 | Reference | 0.12 (-0.09, 0.33) | 0.261 | Reference | 0.02 (-0.17, 0.22) | 0.822 |
| HDL-cholesterol | Reference | 0.01 (-0.08, 0.11) | 0.809 | Reference | 0.11 (0.01, 0.21) | **0.031*** | Reference | -0.07 (-0.16, 0.02) | 0.137 |
| Triglycerides | Reference | -0.04 (-0.18, 0.11) | 0.598 | Reference | -0.10 (-0.25, 0.06) | 0.218 | Reference | 0.17 (0.03, 0.31) | **0.020*** |
| Non-HDL-C | Reference | -0.06 (-0.25, 0.12) | 0.495 | Reference | 0.01 (-0.19, 0.20) | 0.952 | Reference | 0.09 (-0.08, 0.27) | 0.300 |
| TC:HDL ratio | Reference | -0.01 (-0.21, 0.19) | 0.913 | Reference | -0.16 (-0.36, 0.05) | 0.145 | Reference | 0.15 (-0.04, 0.34) | 0.121 |
| Glucose | Reference | 0.02 (-0.13, 0.17) | 0.798 | Reference | -0.02 (-0.17, 0.14) | 0.847 | Reference | -0.06 (-0.21, 0.08) | 0.378 |
| Insulin | Reference | -0.05 (-1.05, 0.94) | 0.920 | Reference | -0.70 (-1.76, 0.36) | 0.194 | Reference | 1.12 (0.16, 2.08) | **0.023*** |
| HOMA-IR | Reference | -0.00 (-0.23, 0.22) | 0.975 | Reference | -0.16 (-0.40, 0.08) | 0.203 | Reference | 0.22 (0.00, 0.44) | **0.049*** |
| Systolic blood pressure | Reference | -1.08 (-3.74, 1.58) | 0.424 | Reference | -0.70 (-3.56, 2.16) | 0.629 | Reference | 0.79 (-1.80, 3.38) | 0.550 |
| Diastolic blood pressure | Reference | -0.64 (-2.58, 1.31) | 0.521 | Reference | -0.42 (-2.51, 1.67) | 0.695 | Reference | 0.64 (-1.25, 2.53) | 0.506 |
| hs-CRP | Reference | -0.95 (-2.18, 0.29) | 0.132 | Reference | -1.47 (-2.79, -0.16) | **0.028*** | Reference | 0.67 (-0.53, 1.88) | 0.271 |
| Combined pre- hypertension/hypertension status ^1^ | Reference | -0.14 (-1.70, 1.42) | 0.860 | Reference | -0.01 (-1.79, 1.77) | 0.993 | Reference | -0.12 (-1.65, 1.40) | 0.874 |

All values are β coefficients and 95% CI unless otherwise specified. ^1^ OR and 95% CI, PDI: plant-based diet index, hPDI: healthy plant-based diet index, uPDI: less healthy plant-based diet index, adjusted for ethnicity, maternal education, smoking status, alcohol intake over the past 12 months, hormonal contraceptive use, and physical activity, energy intake, and diet misreporting status, * indicates results p<0.05.

## Supplementary Table 12: Associations between male plant-based diet quality score trajectory groups and markers of cardiometabolic health at the Gen2-28 year follow-up from the multiple imputation sensitivity analysis (n=256) (number of imputations=20)

|  | **PDI** | | | **hPDI** | | | **uPDI** | | |
| --- | --- | --- | --- | --- | --- | --- | --- | --- | --- |
|  | **Group 1**  **Low** | **Group 2**  **High** | **P value** | **Group 1**  **Low** | **Group 2**  **High** | **P value** | **Group 1**  **Low** | **Group 2**  **High** | **P value** |
| Waist circumference | Reference | -3.35 (-5.71, -0.99) | **0.006*** | Reference | -2.24 (-4.81, 0.33) | 0.087 | Reference | 0.81 (-2.12, 3.73) | 0.587 |
| Waist-to-height ratio | Reference | -0.02 (-0.03, -0.01) | **0.006*** | Reference | -0.01 (-0.03, 0.00) | 0.120 | Reference | 0.01 (-0.01, 0.02) | 0.458 |
| Total cholesterol | Reference | -0.12 (-0.34, 0.09) | 0.254 | Reference | 0.28 (0.05, 0.50) | **0.017*** | Reference | 0.03 (-0.23, 0.30) | 0.793 |
| HDL-cholesterol | Reference | -0.00 (-0.07, 0.07) | 0.994 | Reference | 0.05 (-0.02, 0.13) | 0.155 | Reference | -0.03 (-0.12, 0.05) | 0.406 |
| Triglycerides | Reference | 0.01 (-0.15, 0.17) | 0.919 | Reference | 0.21 (0.04, 0.38) | **0.018*** | Reference | -0.01 (-0.21, 0.18) | 0.898 |
| Non-HDL-C | Reference | -0.15 (-0.36, 0.06) | 0.169 | Reference | 0.19 (-0.03, 0.42) | 0.096 | Reference | 0.06 (-0.20, 0.32) | 0.658 |
| TC:HDL ratio | Reference | -0.15 (-0.43, 0.13) | 0.296 | Reference | 0.08 (-0.22, 0.38) | 0.594 | Reference | 0.17 (-0.17, 0.51) | 0.326 |
| Glucose | Reference | 0.09 (-0.18, 0.37) | 0.502 | Reference | 0.12 (-0.18, 0.42) | 0.435 | Reference | 0.25 (-0.09, 0.58) | 0.154 |
| Insulin | Reference | -0.83 (-2.03, 0.37) | 0.176 | Reference | -0.06 (-1.36, 1.23) | 0.926 | Reference | -0.35 (-1.84, 1.14) | 0.642 |
| HOMA-IR | Reference | -0.19 (-0.46, 0.09) | 0.178 | Reference | 0.01 (-0.29, 0.31) | 0.945 | Reference | -0.07 (-0.41, 0.27) | 0.691 |
| Systolic blood pressure | Reference | -0.37 (-2.79, 2.06 | 0.766 | Reference | -2.45 (-5.03, 0.14) | 0.064 | Reference | 0.23 (-2.73, 3.19) | 0.877 |
| Diastolic blood pressure | Reference | -1.51, (-3.35, 0.33) | 0.108 | Reference | -2.81 (-4.77, -0.85) | **0.005*** | Reference | 0.56 (-1.70, 2.82) | 0.626 |
| hs-CRP | Reference | -0.24 (-0.88, 0.40) | 0.460 | Reference | -0.34 (-1.03, 0.35) | 0.334 | Reference | 0.41 (-0.37, 1.20) | 0.299 |
| Combined pre- hypertension/hypertension status ^1^ | Reference | 0.02 (-1.21, 1.24) | 0.979 | Reference | -0.19 (-1.42, 1.03) | 0.755 | Reference | 0.24 (-1.17, 1.64) | 0.742 |

All values are β coefficients and 95% CI unless otherwise specified. ^1^ OR and 95% CI, PDI: plant-based diet index, hPDI: healthy plant-based diet index, uPDI: less healthy plant-based diet index, adjusted for ethnicity, maternal education, smoking status, alcohol intake over the past 12 months, and physical activity, energy intake, and diet misreporting status, * indicates results p<0.05.

# References:

1. Lachat C, Hawwash D, Ocke MC, Berg C, Forsum E, Hornell A, et al. Strengthening the Reporting of Observational Studies in Epidemiology - nutritional epidemiology (STROBE-nut): An extension of the STROBE statement. Nutr Bull. 2016;41(3):240-51.

2. van de Schoot R, Marit S, D. WS, Sarah D, and Vermunt JK. The GRoLTS-Checklist: Guidelines for Reporting on Latent Trajectory Studies. Structural Equation Modeling: A Multidisciplinary Journal. 2017;24(3):451-67.
